# Prevalence, antibiotic resistance, and virulence gene profiles of uropathogenic *Escherichia coli* (UPEC) isolated from raw milk: implications for public health

**DOI:** 10.1007/s12223-026-01461-x

**Published:** 2026-03-26

**Authors:** Aynur Akan, Seval Cing Yildirim, Cumhur Avsar, Zeynep Yegin

**Affiliations:** 1 Department of Biology, Faculty of Arts & Sciences, Inonu University, Malatya, Turkey; 2 Department of Biology, Faculty of Arts & Sciences, Sinop University, Sinop, Turkey; 3 Medical Laboratory Techniques Program, Vocational School of Health Services, Sinop University, Sinop, Turkey

**Keywords:** Antibiotic resistance, Public health, Raw milk, Urinary tract infection (UTI), Uropathogenic *Escherichia coli* (UPEC), Virulence genes

## Abstract

Raw milk, while nutritionally valuable, may act as a reservoir for zoonotic and antibiotic-resistant microorganisms, creating a potential pathway for foodborne urinary tract infections (FUTIs). Following ingestion, foodborne *Escherichia coli* may colonize the gastrointestinal tract and subsequently reach the urinary tract via the fecal–perineal–urethral route. Among foodborne pathogens, *E. coli* stands out as both a commensal and a versatile pathogen responsible for approximately 80% of uncomplicated urinary tract infections (UTIs). Increasing clinical and epidemiological evidence suggests that food-associated *E. coli* strains carrying uropathogenic traits can contribute to community-acquired UTIs, yet this link remains insufficiently characterized. This study provides a comprehensive assessment of the prevalence, antibiotic resistance, and virulence gene profiles of E. coli isolates obtained from raw milk samples collected in rural areas of Malatya province, Türkiye. A total of 122 raw milk samples were collected, from which 206 bacterial colonies were isolated; 115 isolates were subsequently identified as *E. coli* by phenotypic and biochemical tests. Antibiotic susceptibility analysis revealed complete resistance to cephalothin and notable resistance to ampicillin, tetracycline, and trimethoprim–sulfamethoxazole, yielding a Multiple Antibiotic Resistance (MAR) index of 0.178, indicative of moderate antibiotic selection pressure. Molecular identification via 16 S rRNA sequencing confirmed 51 of 69 isolates (73.91%) as *E. coli* with ≥ 99% similarity. Screening for ten virulence genes demonstrated that Thirty-four of the 51 *E. coli* isolates analyzed for virulence genes (66.60%) carried three or more UPEC-associated virulence determinants, classifying them as potential uropathogenic *E. coli* (UPEC). These findings demonstrate that raw milk can serve not only as a route for *E. coli* contamination but also as a reservoir of multidrug-resistant and uropathogenic strains. The coexistence of antibiotic resistance and UPEC-associated virulence factors in foodborne isolates provides novel evidence linking the food chain to the emergence of FUTIs. Continuous microbiological surveillance, antibiotic stewardship, and strict hygiene protocols throughout the dairy production chain are essential to prevent foodborne urinary tract infections and protect public health, directly supporting the United Nations Sustainable Development Goals related to good health and well-being (SDG 3) and responsible consumption and production (SDG 12).

**Supplementary Information:**

The online version contains supplementary material available at https://doi.org/10.1007/s12223-026-01461-x.

## Introduction

Milk is a nutritionally rich food that contributes to bone health, immune function, and overall metabolic regulation (Heaney 2000). Owing to its complex composition, milk also supports a diverse microbial community, which may influence both health benefits and microbiological safety (Quigley et al. 2013; Zhang et al. 2021). Despite the nutritional value of milk, systematic surveillance data on pathogenic *E. coli*, particularly uropathogenic *E. coli* (UPEC), in food matrices remain limited.

In addition to these benefits, milk also provides a favorable environment for microbial growth, particularly for psychrotrophic bacteria; therefore, strict hygiene controls are essential during production and processing (Yalew et al. 2024). Among the bacterial contaminants, *E. coli* is one of the most frequently encountered foodborne pathogens, commonly linked to raw and undercooked meat, poultry, raw milk, and untreated water (Bielecki 2003). *E. coli* represents a remarkable transition between commensalism and pathogenicity and, as a versatile pathogen, is responsible for more than two million human deaths annually through both intestinal and extraintestinal infections (Tenaillon et al. 2010).

Beyond its presence in food, pathogenic *E. coli* is a zoonotic and versatile foodborne pathogen possessing numerous virulence genes that regulate diverse cellular processes and determine its pathogenic potential (Kaper et al. 2004; Li et al. 2022). Based on infection site, *E. coli* strains are broadly classified into two groups: intestinal pathogenic *E. coli* (IPEC), which cause gastrointestinal infections, and extraintestinal pathogenic *E. coli* (ExPEC), which cause infections outside the intestine. The ExPEC group includes several clinically important pathotypes, such as uropathogenic *E. coli* (UPEC), neonatal meningitis *E. coli* (NMEC), septicemia-associated *E. coli* (SEPEC), avian pathogenic *E. coli* (APEC), and mammary pathogenic *E. coli* (MPEC) (Sora et al. 2021).

ExPEC strains are the predominant causative agents of urinary tract infections (UTIs), accounting for nearly 90% of community-acquired cases and most pyelonephritis episodes. Increasing molecular and epidemiological evidence indicates that ExPEC commonly originates from the intestinal microbiota, where it establishes persistent colonization prior to ascending infection of the urinary tract. Following oral exposure through contaminated food products, ExPEC can survive within the gastrointestinal tract and subsequently disseminate to the urinary system via the fecal–perineal–urethral route, a mechanism consistently observed in community-acquired UTIs (Manges et al. 2001; Vincent et al. 2010; Nordstrom et al. 2013; Liu et al. 2018). Recent whole-genome sequencing and One Health–oriented studies have further strengthened the evidence for interconnection between food animal reservoirs and human UTI–associated ExPEC lineages by demonstrating shared sequence types, virulence determinants, and antimicrobial resistance profiles among isolates recovered from retail poultry, food animals, and community-acquired UTI cases (Liu et al. 2018; Manges et al. 2019; Guragain et al. 2025). These findings support the hypothesis that foodborne exposure contributes to intestinal colonization by ExPEC strains capable of subsequent ascending urinary tract infection. Within this broader transmission framework, although direct epidemiological evidence specifically linking raw milk consumption to UTIs remains limited, the detection of antimicrobial-resistant and virulence-associated *E. coli* in raw milk is consistent with this foodborne urinary tract infection (FUTI) paradigm.

Other urinary tract diseases, such as prostatitis and catheter-associated UTIs, are also frequently linked to ExPEC infections (Dale and Woodford 2015; Sora et al. 2021). The majority of UTIs in young, otherwise healthy women are attributed to ExPEC, largely due to anatomical and physiological factors including a shorter urethra, closer proximity of the urethral opening to the perianal region, and hormonal influences that facilitate ascending bacterial colonization (Flores-Mireles et al. 2015; Poolman and Wacker 2016; García et al. 2023). Recent genomic and epidemiological studies have demonstrated that ingestion of foodborne ExPEC strains can promote intestinal colonization by uropathogenic lineages capable of subsequent ascending infection (Manges et al. 2001, 2019; Liu et al. 2018; Guragain et al. 2025). Although direct clinical evidence specifically linking raw milk consumption to increased UTI incidence in women remains limited, the detection of antimicrobial-resistant and virulence-associated *E. coli* in raw milk supports its potential role within this broader foodborne transmission framework.

Since the early 2000 s, ExPEC has also emerged as a major contributor to antibiotic resistance, particularly against cephalosporins and fluoroquinolones (Pitout 2012). At the molecular level, *E. coli* strains that cause urinary tract infections differ markedly from commensal isolates and are referred to as uropathogenic *E. coli* (UPEC) (Foxman and Brown 2003). UPEC is the predominant etiological agent in both uncomplicated and complicated UTIs, and infection with UPEC increases the likelihood of recurrence within six months (Medina and Castillo-Pino 2019). It accounts for approximately 75% of uncomplicated and more than half of complicated UTI cases, particularly affecting women and older adults (Zhou et al. 2023; Whelan et al. 2023).

UPEC employs a broad repertoire of virulence and fitness determinants to colonize, survive, and disseminate within the urinary tract (Subashchandrabose and Mobley 2015). These include fimbrial and non-fimbrial adhesins, flagella, toxins, lipopolysaccharides, polysaccharide capsules, outer-membrane vesicles, and iron-acquisition systems (Zhou et al. 2023). The primary target organs are the urethra, bladder, and kidneys, and severe infections may progress to bacteremia, septicemia, or urosepsis (Zhou et al. 2023). Biofilm formation by UPEC enhances bladder colonization and markedly increases antimicrobial resistance (Klein and Hultgren 2020).

UPEC-associated virulence factors can be classified by function: adherence-related (type 1, P, S, and F1C pili; Dr adhesins), toxins (HlyA, CNF1), immune-evasion factors (capsular antigens, CNF1, Yersiniabactin), and iron-uptake systems (Aerobactin, Enterobactin, Salmochelin, Yersiniabactin), as well as additional surface molecules such as Antigen 43 and flagella (Ulett et al. 2007; Schwan 2008; Nielubowicz and Mobley 2010; Chaturvedi et al. 2012; Flores-Mireles et al. 2015). Isolates carrying three or more virulence genes are generally defined as potential UPEC strains (Spurbeck et al. 2012; García-Meniño et al. 2022; García et al. 2023). Combinations of these genes influence infection severity, therapeutic response, and recurrence, although disease outcome also depends on host-related factors such as immune status, age, hormonal balance, and environmental conditions (Köves and Wullt 2016).

Foodborne urinary tract infections (FUTIs) represent an emerging paradigm distinct from classical foodborne illnesses, highlighting the role of the food chain as a reservoir for extraintestinal pathogenic *Escherichia coli* (ExPEC) capable of causing urinary tract infections. Unlike conventional foodborne diseases that primarily involve gastrointestinal symptoms, FUTIs result from intestinal colonization by uropathogenic strains followed by ascending infection of the urinary tract. Recent One Health–oriented genomic and functional studies have provided compelling evidence supporting this transmission pathway. Comparative genomic analyses have demonstrated close genetic relatedness and shared virulence profiles between ExPEC isolates recovered from retail meat—particularly poultry—and human UTI cases. Moreover, poultry-derived ExPEC strains have been shown to exhibit strong adhesion to bladder epithelial cells and robust biofilm-forming capacity, key pathogenic mechanisms required for urinary tract colonization and persistence. These findings substantiate that foodborne ExPEC are not merely contaminants but possess functional uropathogenic potential comparable to clinical UPEC isolates (García et al. 2023). The FUTI mechanism is schematically illustrated in Supplementary Figure S1.

Foodborne urinary tract infections (FUTIs) represent a clinically significant transmission pathway in which zoonotic extraintestinal pathogenic *Escherichia coli* (ExPEC) strains originating from food animal reservoirs contribute to community-acquired UTIs. Large-scale genomic source attribution studies have provided direct clinical evidence supporting this paradigm, demonstrating that approximately 17–18% of human UTI isolates are of zoonotic origin, primarily linked to retail meat products (Aziz et al. 2025). Poultry has been identified as the dominant reservoir, with zoonotic ExPEC-associated sequence types most frequently recovered from retail chicken and turkey products, highlighting the central role of poultry consumption in foodborne urinary transmission. Notably, zoonotic ExPEC-related UTIs occur at significantly higher rates in women compared to men (approximately 19.7% versus 8.5%), underscoring sex-specific vulnerability within this transmission pathway (Aziz et al. 2024, 2025).

Complementary molecular and epidemiological investigations further demonstrate close phylogenetic overlap between ExPEC strains isolated from retail meats and those causing human UTIs, sharing common virulence profiles and antimicrobial resistance determinants (Johnson et al. 2005; Jakobsen et al. 2010; Vincent et al. 2010; Liu et al. 2018; Manges et al. 2019; Guragain et al. 2025). Collectively, these findings provide robust clinical and mechanistic evidence that consumption of contaminated meat products—particularly poultry—facilitates intestinal colonization by uropathogenic lineages capable of subsequent ascending urinary tract infection, firmly establishing the FUTI paradigm.

Increasing evidence indicates that the food chain contributes substantially to the growing prevalence of antibiotic-resistant UTIs in the community. Uropathogenic *E. coli* (UPEC) strains have been repeatedly detected in retail meat products, particularly poultry, suggesting a potential link between foodborne exposure and extraintestinal infections (Jakobsen et al. 2010). The incidence of UTIs varies across regions depending on food production practices, antibiotic usage, and hygiene conditions, yet the overall trend reveals a continuous global increase (Priyanka et al. 2023).

In the present study, a combination of microbiological and molecular techniques was employed to identify and characterize *Escherichia coli* strains isolated from raw milk samples. These complementary methods provided detailed information on species identity, phenotypic traits, antibiotic susceptibility, and virulence gene profiles. Colony morphology was first evaluated on selective and differential media, followed by biochemical confirmation using IMViC tests. Antimicrobial susceptibility was determined using the Kirby–Bauer disk diffusion method, while molecular tools—including PCR and 16 S rRNA gene sequencing—were applied for accurate species identification and detection of virulence-associated genes.

This integrated approach enabled a comprehensive assessment of *E. coli* prevalence, antibiotic resistance, and virulence characteristics, thereby supporting the identification of potential uropathogenic *E. coli* (UPEC) strains in raw milk. Unlike previous studies that focused solely on either antibiotic resistance or virulence profiling, our work combines both datasets to reveal the coexistence of multidrug resistance and UPEC-related virulence determinants in a food matrix. This provides novel evidence for the potential role of raw milk as a reservoir of foodborne uropathogens. By integrating molecular and phenotypic data, this study establishes a new framework for detecting and assessing uropathogenic *E. coli* in food matrices, emphasizing their relevance to antimicrobial resistance surveillance and public health monitoring, and providing a foundation for future risk assessment studies on foodborne uropathogens.

## Materials and methods

### Collection of raw milk samples

A total of 122 raw milk samples were collected from Malatya Province between May 23, 2024, and October 14, 2024. The study was conducted in this region due to the lack of available data on the presence of uropathogenic *Escherichia coli* (UPEC) in raw milk and its potential role in foodborne urinary tract infections. The sampling strategy was further informed by previous findings from the same region, including a published study on traditionally produced white cheese, which highlighted challenges related to microbial safety in local dairy products and raised the possibility that raw milk used in traditional cheese production could serve as a potential source of contamination (Cing Yildirim and Ates 2022).

**Fig. 1:**
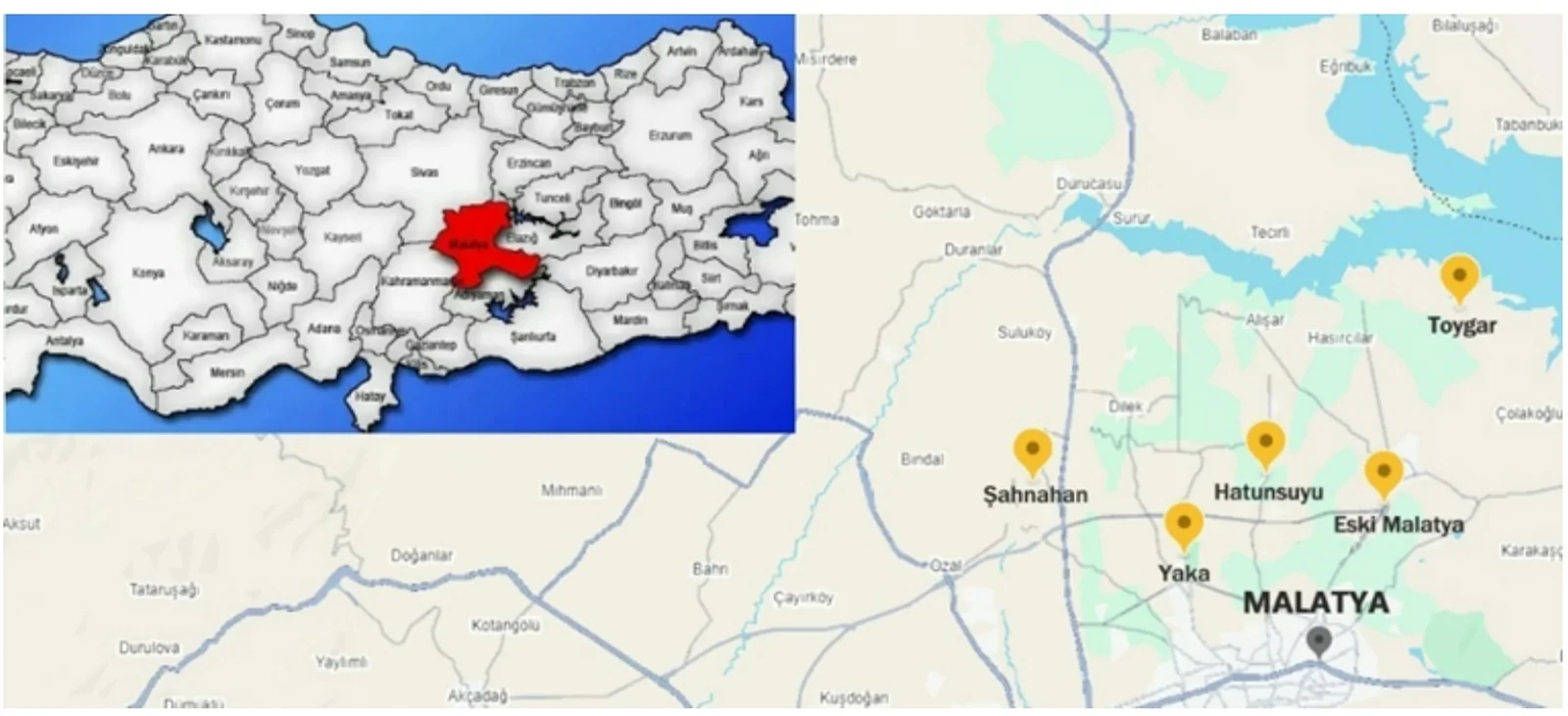
Geographical locations of raw milk sampling from five selected regions of Malatya, Turkey

Milk samples were collected directly from local producers operating in rural settlements across the Toygar, Hatunsuyu–Mahmutlu, Şahnahan, Yaka, and Eski Malatya regions (Fig. 1) to reflect common small-scale production and handling practices within the province. A target sample size exceeding 100 samples was set to ensure sufficient diversity and regional representativeness. Each approximately 50 mL milk sample was placed in sterile transport containers and transported to the Industrial Biotechnology Research Laboratory under cold chain conditions (4 ± 1 °C). Pre-analysis preparation of the samples was performed on the same day.

### Preliminary identification of *E. coli* by cultural methods

Cultural methods are a fundamental step in the preliminary identification of bacteria. In this step, preliminary information about the possible presence of *E. coli* was obtained by evaluating the colony morphology of the bacteria on selective and/or differential media. McConkey (MAC) agar, Nutrient Agar (NA), and Eosin Methylene Blue (EMB) agar were used in this study; each medium allowed the differentiation of specific phenotypic characteristics of *E. coli*. MAC agar provided differentiation based on lactose fermentation, while NA was used for pure colony isolation. EMB agar, on the other hand, produced the characteristic metallic green highlights of *E. coli*, enabling its differentiation from other bacteria.

Raw milk samples obtained from different geographical regions were delivered to the laboratory under cold chain conditions as quickly as possible. For enrichment, 1 mL of each raw milk sample was inoculated into 9 mL of sterile peptone water. This mixture was incubated at 37 °C in a shaking incubator at 110 rpm for 18–24 h. Additionally, 100 µL of raw milk samples were directly inoculated onto MAC medium using the spread plate method, and the plates were incubated at 37 °C for 18–24 h. At the end of incubation, serial dilutions were applied to enriched raw milk samples based on bacterial growth densities on the MAC agar medium. To determine the total bacterial count in the samples as individual colonies, sequential dilutions were prepared with 0.9% (w/v) physiological saline (PS) solution. For this purpose, 1 mL of each milk sample was mixed with 9 mL of PS at a 1:10 ratio, resulting in dilutions of 10^− 4^−10^− 7^. 0.1 mL of each dilution was taken and inoculated onto MAC agar using the spread plate method under sterile conditions. After inoculation, the plates were incubated at 37 °C for 18–24 h.

Pink-red lactose-fermenting bacterial colonies observed on MAC agar were identified using a colony counter. The identified colonies were picked using a sterile needle and loop and inoculated onto EMB agar plates by streaking. The prepared plates were incubated at 37 °C for 24 h. On EMB agar, colonies with the characteristic metallic, bright green color of *E. coli* were observed. Samples from these metallic colonies were taken with a sterile loop and inoculated onto NA for single colony formation. The inoculated plates were incubated at 37 °C for 18–24 h to obtain pure colonies.

After inoculation on NA, six of the individual colonies formed after 24–48 h of incubation at 37 °C were taken with a needle loop and inoculated onto EMB agar using the streak method for further testing of pure cultures. The inoculated plates were incubated at 37 °C for 18–24 h. This method allowed the characteristic colony morphology of bacteria to be observed more clearly on EMB agar.

Colonies showing typical growth on EMB agar were subsequently transferred into Brain Heart Infusion (BHI) broth for further analyses. For definitive confirmation, samples were taken from a minimum of one and a maximum of three representative colonies per milk sample, ensuring consistency and reproducibility of isolate selection.

### Characterization of *E. coli* isolates

All stock cultures were removed from the − 80 °C freezer and thawed in a container containing crushed ice to enable characterization of *E. coli* or other Enterobacteriacea members. Completely thawed samples (200 µL) were inoculated into 20 mL of previously prepared Mueller Hinton Broth (MHB). These cultures, prepared at approximately 1% (v/v), were incubated at 37 °C and 110 rpm in a shaking incubator for 18–24 h. Following the incubation period, biochemical tests were performed to characterize isolates suspected of being *E. coli*. Biochemical characterization of the isolates was performed using standard microbiological procedures. Gram staining was applied to assess cell morphology and Gram reaction. Catalase activity was determined by observing oxygen bubble formation following the application of 3% hydrogen peroxide to bacterial colonies, while oxidase activity was evaluated using a commercial oxidase test reagent. Triple Sugar Iron (TSI) agar was used to assess carbohydrate fermentation patterns, gas production, and hydrogen sulfide (H_2_S) formation. Indole production was tested using Kovac’s reagent after incubation in tryptophan broth. Methyl red (MR) and Voges–Proskauer (VP) tests were conducted to evaluate glucose fermentation pathways, and citrate utilization was assessed using Simmons citrate agar. All tests were performed using standardized protocols, and results were interpreted according to established microbiological criteria.

### Antibiotic susceptibility testing

Antibiogram test was performed on 120 different bacterial samples, which were identified as *E. coli* with biochemical tests. Kirby-Bauer disk diffusion test was used to determine the antibiotic susceptibility profiles of *E. coli* strains. Mueller-Hinton Agar (MHA) solid medium was used for testing cultures. 10% (v/v) culture was established by adding 200 µL of completely dissolved stock bacteria samples to 20 mL of prepared MHB. The prepared mixture was incubated at 37 °C in a shaking incubator at 110 rpm for 18–24 h. Following incubation, 100 µL of stock bacterial cultures diluted to a 0.5 McFarland standard were spread homogeneously onto the surface of the MHA using a Drigalski stick. A total of seven separate MHA plates were labeled for each bacterial culture. Three different antibiotic discs were placed on the first five of these plates, and two different antibiotic discs were placed on the remaining two plates, at distances adjusted according to CLSI (Clinical and Laboratory Standards Institute), 2022 standards. A total of 19 different antibiotics were used to determine the antibiotic susceptibility profile of each bacterial culture. Information on the antibiotic discs used in this study is presented in Table 1, which summarizes the classes, subclasses, and disk concentrations of the antibiotics tested to ensure methodological transparency and reproducibility of the antimicrobial susceptibility assays. After disc placement, the plates were incubated at 37 °C for 24 h. Following incubation, inhibition zone diameters were measured using an electronic caliper and interpreted according to CLSI guidelines (2022). All assays were performed under standardized conditions, and ambiguous or borderline results were re-evaluated to ensure measurement accuracy. Following antimicrobial susceptibility testing, a subset of isolates was selected for virulence gene analysis. Isolates exhibiting identical or highly similar antibiotic inhibition zone profiles were considered phenotypically redundant and were therefore excluded to avoid overrepresentation of closely related isolates. This selection strategy was applied to ensure diversity among the tested isolates while maintaining representativeness of the overall isolate collection. As a result, 69 non-redundant isolates were subjected to virulence gene screening. Although genomic DNA was extracted from all isolates, virulence gene analysis was performed on a representative subset to ensure analytical efficiency while preserving phenotypic diversity.

**Table 1:** Classes, subclasses, and disk concentrations of antibiotics used in this study

Antibiotic class	Subclass	Antibiotic name/Symbol	Concentration of disc (µg)
Penicillins	Aminopenicillin	Ampicillin (AMP/AM)	10
β-lactam/β-lactamase inhibitor combinations		Piperacillin/tazobactam (PPT/TPZ)	100/10
		Ampicillin/sulbactam (ASB/SAM)	10/10
		Amoxicillin/clavulanic acid (AMC/AMC)	20/10
Cephalosporins	I	Cephalotin (CFL/KF)	30
	II	Cefuroxime (CRX/CXM)	30
	III	Cefotaxime (CTX/CTX)	30
	IV	Cefepime (CPM/FEP)	30
Penems	Carbapenem	Ertapenem (ETP/ETP)	10
	Carbapenem	Meropenem (MER/MEM)	10
Aminoglycosides		Gentamicin (GEN/CN)	10
		Amikacin (AMI/AK)	30
Quinolones	Quinolone I	Nalidixic acid (NAL/NA)	30
	Fluoroquinolone	Norfloxacin (NOR/NOR)	10
	Fluoroquinolone	Ciprofloxacin (CIP/CIP)	5
Folate pathway inhibitors		Trimethoprim/sulfamethoxazole (SUT/SXT)	1.25/23.75
Other antibiotics		Fosfomycin (FOS/FF)	200
	Nitrofuran	Nitrofurantoin (NIT/F)	300

### Selection and functional categorization of virulence genes

In order to comprehensively assess the pathogenic potential of *E. coli* isolates recovered from raw milk, a targeted panel of virulence-associated genes was selected based on predefined functional and pathogenic criteria relevant to uropathogenic *E. coli* (UPEC). The selected genes were chosen to represent major virulence categories involved in UPEC pathogenesis, including adhesion to host tissues (fimH, papC), toxin-mediated host damage (hlyA, cnf1), iron acquisition systems (fyuA, iroN, iutA), capsule formation (kpsMII), and biofilm formation and persistence (agn43, vat). Although the *vat* gene is not commonly detected in UPEC isolates, it was included in the panel due to its association with extraintestinal pathogenic *E. coli* and its relevance for evaluating zoonotic and foodborne transmission potential Detailed information on the functional roles and virulence categories of each gene is provided in Supplementary Table S2. Genomic DNA was extracted from all isolates; however, virulence gene analysis was performed on a representative subset to ensure analytical efficiency while preserving phenotypic diversity.

### Genomic DNA isolation and identification of UPEC virulence genes with PCR

For DNA isolation, stock bacterial cultures kept in 85% glycerol solution (v/v) were removed from the freezer and thawed in a container filled with crushed ice. Completely thawed samples (200 µL) were inoculated with 20 mL of previously prepared MHB. The culture prepared at 10% (v/v) was incubated at 37 °C and 110 rpm for 18–24 h. After incubation, DNA isolation of isolates suspected to be *E. coli* was performed using PureLink Genomic DNA Mini Kit (Thermo Fisher Scientific, Waltham, MA, USA). As shown in Online Resource 1 (Supplementary Figure S2), the uncropped gel image demonstrates the genomic DNA integrity of *E. coli* isolates prior to PCR amplification, confirming that the extracted DNA samples were intact and suitable for downstream molecular analyses.

In order to evaluate the uropathogenic potential of *E. coli* isolates, PCR reactions of isolated bacterial genomic DNA samples were performed for the selected UPEC virulence genes. Based on the evaluation of bacterial samples’ antibiogram test results according to CLSI standards, only 69 of 120 genomic DNA samples were screneed for UPEC virulence genes. Bacterial samples with similar inhibition zone values were excluded from further analysis. PCR reactions were performed using specific primers for target UPEC virulence genes. The primer sequences and expected amplicon sizes used for PCR amplification of UPEC-associated virulence genes are provided in Table 2. First, PCR amplification was performed for *fimA* virulence gene analysis. A total of 25 µl of PCR mixture included 2 mM MgCl_2_, 1x Taq buffer, 0.2 mM dNTP mix, 0.5 µM forward and reverse primer pair, 1.5 U Taq polymerase (Thermo Fisher Scientific, Waltham, MA, USA) and 1 µl *E. coli* DNA. Thermal cycler conditions were as follows: 35 cycles of 40 s at 94 °C, annealing for 45 s at 60 °C and extension for 60 s at 72 °C. A predenaturation step for 6 min at 95 °C and a final extension step for 12 min at 72 °C were included. The similar PCR and thermal cycler conditions were used for the other investigated virulence genes with a difference in annealing temperatures. In PCR protocols, a 40-second annealing time at 60 °C was applied for most of the investigated genes, but for *iroN*, *kpsMII*, *agn43* and *iutA* genes, this temperature was optimized as 58 °C. Fragments were run on 2.5% agarose gels stained with ethidium bromide by electrophoresis and analyzed with gel documentation system (SYNGENE Ingenius 3, England) to confirm the expected fragment sizes. 50 bp DNA Ladder RTU (Ready-to-Use) (GeneDireX, Inc., Taiwan) was used in the experiments. PCR assays were performed in duplicate to ensure technical reproducibility. Negative controls (no-template controls) were included in each PCR run to monitor potential contamination. Amplification products were evaluated based on expected amplicon sizes. Each DNA isolate was separately analyzed for the presence of 10 different targeted virulence genes. According to the widely accepted criterion stated in the introduction part, the status of UPEC was assigned to the isolates positive for ≥ 3 of the virulence genes.

**Table 2:** Primers used for PCR amplification of UPEC-associated virulence genes (Primer sequences are shown in the 5′–3′ direction)

Virulence gene	Primer sequence (5′–3′)	Amplicon size (bp)
hlyA	F: AACAAGGATAAGCACTGTTCT R: ACCATATAAGCGGTCATTCCC	1177
papC	F: GACGGCTGTACTGCAGGGTGTGGCG R: ATATCCTTTCTGCAGGGATGCAATA	328
cnf1	F: AAGATGGAGTTTCCTATGCAGGAG R: CATTCAGAGTCCTGCCCTCATTATT	498
fimA	F: GTTGTTCTGTCGGCTCTGTC R: ATGGTGTTGGTTCCGTTATTC	447
fyuA	F: TGATTAACCCCGCGACGGGAA R: CGCAGTAGGCACGATGTTGTA	785
vat	F: AGAGACGAGACTGTATTTGC R: GTCAGGTCAGTAACGAGCAC	289
iroN	F: AAGTCAAAGCAGGGGTTGCCCG R: GACGCCGACATTAAGACGCAG	667
kpsMII	F: GCGCATTTGCTGATACTGTTG R: AGGTAGTTCAGACTCACACCT	578
agn43	F: CTGGAAACCGGTCTGCCCTT R: CCTGAACGCCCAGGGTGATA	433
iutA	F: GGCTGGACATCATGGGAACTGG R: CGTCGGGAACGGGTAGAATCG	302

### 16 S rRNA analysis

The same number of bacterial genomic DNA samples (n = 69) analyzed for UPEC virulence genes were prepared for sequencing by applying PCR for 16S rRNA gene. PCR reaction was carried out in 50 µl total volume including 1x PCR buffer, 0.1 mM dNTP mix, 0.4 µM forward (27F: 5′-AGAGTTTGATCMTGGCTCAG-3’) and reverse (1492R: 5′-TACGGYTACCTTGTTACG ACTT-3’) universal primer pair, and 5 U Taq DNA polymerase (Thermo Fisher Scientific, Waltham, MA, USA). Thermal cycler (Techne TC-5000, California, USA) conditions for 16 S rRNA analysis were as follows: 35 cycles of 60 s at 95 °C, annealing for 60 s at 63 °C and extension for 60 s at 72 °C. A predenaturation step for 5 min at 95 °C and a final extension step for 10 min at 72 °C were included. PCR products were run on 1.5% agarose gels stained with ethidium bromide by electrophoresis and analyzed with gel documentation system (SYNGENE Ingenius 3, England). Amplicons that gave a single and dominant band with primers specific to the 16 S rRNA gene were sent to the laboratory of AQUATAYF Biotechnology AR-GE Industry and Trade Ltd. Co. under cold chain conditions for sequence analysis.

### Statistical and bioinformatics analyses

Correlation analysis between antibiogram and UPEC-associated genes was performed using the Past 4.03 statistical analysis program. For this purpose, resistance and intermediate susceptibility of the strains were assigned a value of ‘1’, and susceptibility was assigned a value of ‘0’. Additionally, the presence of the relevant gene regions in the PCR screenings was evaluated as ‘1’, and their absence was evaluated as ‘0’. Pearson correlation was applied for the analysis and significance levels were determined at *p* < 0.05 level.

The 16 S rRNA PCR products obtained in the study were sequenced by Sanger sequencing method and the obtained sequence data were compared with the NCBI database by BLAST (Basic Local Alignment Search Tool) analysis. The phylogenetic tree was constructed using the maximum likelihood method and the Tamura-Nei (1993) model. The tree with the highest log-likelihood value (−8.278.16) is presented. The starting tree for the heuristic search was selected by selecting the tree with the highest log-likelihood from among the trees obtained using the Neighbor-Joining (NJ) method (Saitou and Nei 1987) and Maximum Parsimony (MP) methods. The NJ tree was constructed based on the pairwise distance matrix calculated using the Tamura-Nei (1993) model. The MP trees were selected as the tree with the shortest length among analyses conducted with 10 different random starting trees. The proportion of positions in the tree where at least one base could be unambiguously identified is indicated for each internal node. The analysis was performed with 101 nucleotide sequences covering 972 positions. Evolutionary analyses were conducted using the MEGA12 software, supporting up to 3 parallel threads (Kumar et al. 2024; Tamura and Nei 1993; Saitou and Nei 1987).

All experimental procedures were performed using standardized protocols to ensure consistency and reproducibility across analyses.

## Results

**Fig. 2:**
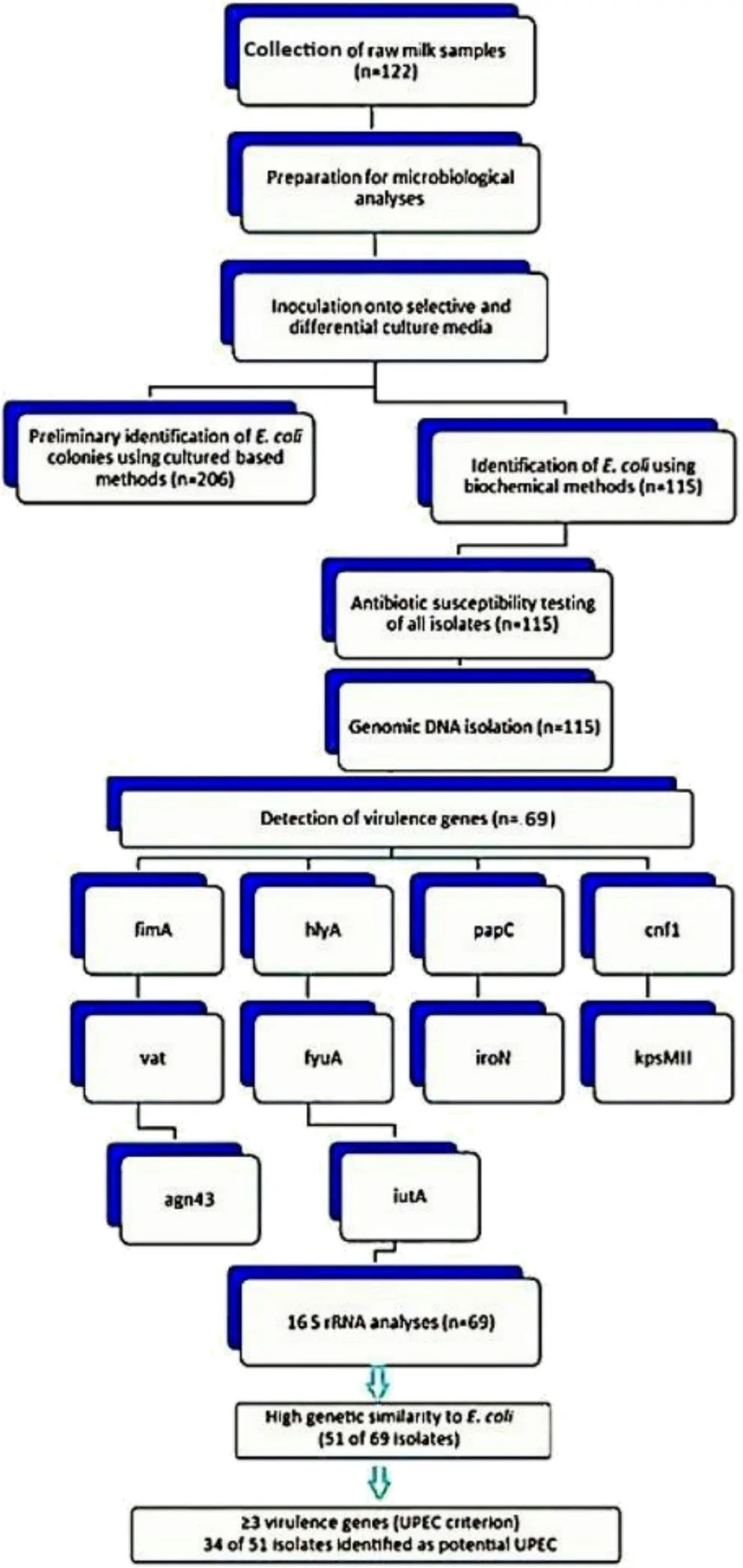
Schematic overview of the study workflow and key findings. Raw milk samples (*n* = 122) were collected and subjected to microbiological analyses. Following culture-based screening, 206 presumptive *Escherichia coli* colonies were obtained, of which 115 isolates were confirmed by biochemical identification. All confirmed isolates were tested for antibiotic susceptibility and screened for UPEC-associated virulence genes. A subset of isolates (*n* = 69) was further analyzed by 16 S rRNA gene sequencing, confirming high genetic similarity to *E. coli* in 51 isolates. Based on the presence of three or more virulence genes, 34 of these isolates were classified as potential uropathogenic *E. coli* (UPEC)

Figure 2 provides a schematic overview of the study workflow and key outcomes, summarizing the progression from raw milk sampling to the identification of potential uropathogenic *Escherichia coli* (UPEC) isolates. A total of 122 raw milk samples were collected and subjected to microbiological and molecular analyses. Following culture-based screening and biochemical identification, 115 *E. coli* isolates were obtained and evaluated for antibiotic susceptibility and UPEC-associated virulence genes. A subset of isolates was further confirmed by 16 S rRNA gene sequencing, enabling the classification of isolates with uropathogenic potential. The detailed results of each analytical step are presented below.

### Findings of identification of *E. coli* by cultural and biochemical methods

A total of 122 raw milk samples were analyzed using culture-based microbiological methods. Samples were first inoculated on selective MAC agar medium. Red/pink colonies from MAC agar were transferred to EMB agar. After incubation, colonies that developed a characteristic metallic highlight on EMB agar were evaluated for preliminary identification of *E. coli*. Based on these preliminary evaluation criteria, a total of 206 colonies likely to be *E. coli* were obtained from 73 different milk samples. Pure cultures were established by selecting at least one and at most three colonies from each milk sample. Furthermore, 49 milk samples with colonies not displaying metallic highlights on EMB agar were excluded from further analysis since they did not meet the preliminary diagnostic criteria for *E. coli*. Based on the preliminary evaluation criteria, 59.8% of the collected milk samples were found to contain *E. coli* or other *Enterobacter* species.

IMVIC tests and other basic biochemical tests were applied to 206 colonies isolated from 73 raw milk samples. According to the IMVIC test results, 115 colonies obtained from 47 milk samples showed the typical biochemical profile specific to *E. coli* (Indole: positive, Methyl Red: positive, Voges-Proskauer: negative, Citrate: negative). In addition, all isolates were determined to be catalase positive and oxidase negative. The TSI test revealed the typical acid/acid reaction and gas production in the isolates. Microscopic examinations using Gram staining determined that the isolates had Gram-negative rod morphology. These findings suggest that a significant portion of the bacteria isolated from raw milk samples are biochemically compatible with *E. coli*. The remaining 91 colonies showed incompatible results with this biochemical profile and they might belong to enteric bacteria other than *E. coli*. In our study, IMVIC positivity rate covers approximately 64% (47/73) of milk samples and 55.8% (115/206) of the colonies. Elimination of colonies which give negative IMVIC results before molecular identification increases the specificity of the test and reduces the likelihood of false-positive diagnoses.

### Antibiotic resistance profiles and MAR index analysis

In the study, antibiotic resistance and susceptibility profiles of 115 isolates biochemically identified as *E. coli* were determined. Nineteen antibiotics were tested as previously mentioned in materials and methods section. All of the tested *E. coli* isolates were resistant to cephalothin. Varying degrees of resistance were observed to other antibiotics. The resistance rates were determined as follows: 0% (*n* = 0) norfloxacin, 0.86% (*n* = 1) fosfomycin, 1.73% (*n* = 2) cefepime, 2.6% (*n* = 3) piperacillin, 3.47% (*n* = 4) cefuroxime, 3.47% (*n* = 4) nitrofurantoin, 4.34% (*n* = 5) amikacin, 5.21% (*n* = 6) ciprofloxacin, 6.08% (*n* = 7) ertapenem, 6.08% (*n* = 7) amoxicillin, 6.08% (*n* = 7) gentamicin, 6.08% (*n* = 7) nalidixic acid, 6.95% (*n* = 8) cefotaxime, 8.69% (*n* = 10) sulfamethoxazole, 25.21% (*n* = 29) ampicillin. When antibiotic sensitivity rates were examined, it was found that the majority of the isolates showed high sensitivity to many antibiotics. The highest susceptibility rates were observed as follows: 99.13% (*n* = 114) fosfomycin, 96.52% (*n* = 111) ceftazidime and meropenem, 94.78% (*n* = 109) ampicillin, amikacin and cefuroxime, 93.91% (*n* = 108) ertapenem and ciprofloxacin, 93.04% (*n* = 107) amoxicillin and gentamicin, 91.3% (*n* = 105) nalidixic acid and sulfamethoxazole, 90.43% (*n* = 104) cefotaxime, 88.69% (*n* = 102) cefepime, 87.82% (*n* = 101) nitrofurantoin and norfloxacin, and 86.95% (*n* = 100) piperacillin. These data indicate that a significant portion of the isolates are still susceptible to broad-spectrum antibiotics. Resistance and susceptibility profiles of the isolates to various antibiotics are depicted in Table 3.

**Table 3:** Resistance and susceptibility profiles of the isolates to various antibiotics

Antibiotic	Sensitive	Intermediate	Resistant	Positivity (%)*	Negativity (%)*
1.AMP (AM-10)	85	1	29	73,9	25,21
2.AMI (AK-30)	109	1	5	94,78	4,34
3.AMC (AMC-30)	107	1	7	93,04	6,08
4.ASB (SAM-20)	109	3	3	94,78	2,6
5.CPM (FEP-30)	102	1	2	88,69	1,73
6.CTX (CTX-30)	104	3	8	90,43	6,95
7.CAZ (CAZ-30)	111	0	4	96,52	3,47
8.CRX (CXM-30)	109	2	4	94,78	3,47
9.CFL (KF-30)	0	0	115	0	100
10.CIP (CIP-5)	108	1	6	93,91	5,21
11.ETP (ETP-10)	108	0	7	93,91	6,08
12.FOS (FF-200)	114	0	1	99,13	0,86
13.GEN (CN-10)	107	1	7	93,04	6,08
14.MER (MEM-10)	111	2	2	96,52	1,73
15.NAL (NA-30)	105	3	7	91,3	6,08
16.NIT (F-300)	101	0	4	87,82	3,47
17.NOR (NOR-10)	101	4	0	87,82	0
18.PPT (TPZ-110)	100	2	3	86,95	2,6
19.SUT (SXT-25)	105	0	10	91,3	8,69

*The positivity rate represents the total percentage of isolates susceptible and intermediate to the antibiotic, while the negativity rate represents the percentage of the resistant isolates. These two rates provide a comparative analysis for evaluating antibiotic efficacy and determining the distribution of resistance

**Fig. 3:**
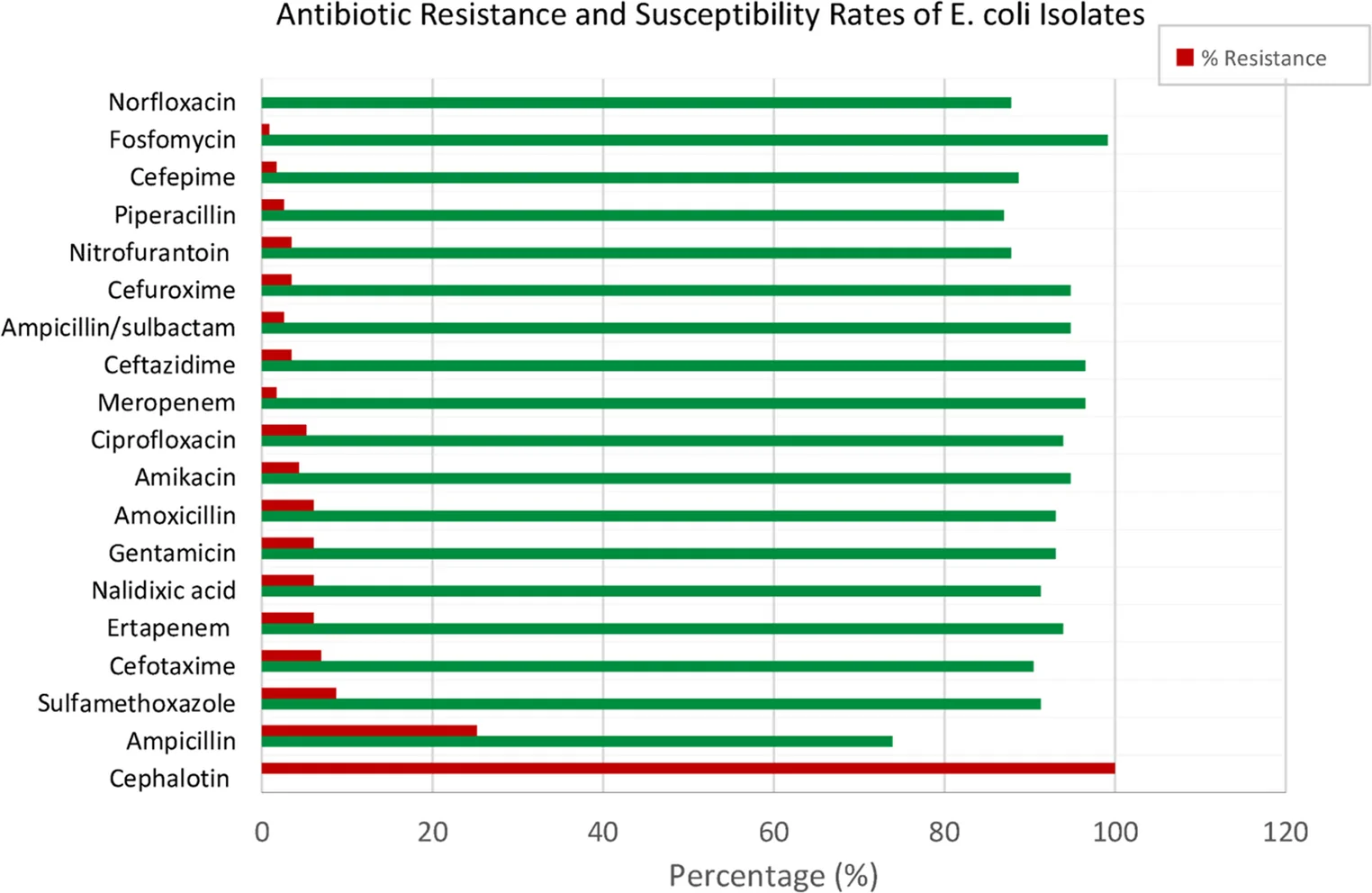
Resistance (red) and susceptibility (green) rates of *Escherichia coli* strains isolated from raw milk samples against different antibiotics. The graph summarizes the antimicrobial susceptibility profiles of 115 isolates tested against 19 antibiotic agents representing multiple antimicrobial classes. Values are expressed as percentages, illustrating the overall distribution of resistant and susceptible phenotypes among the tested isolates

**Fig. 4:**
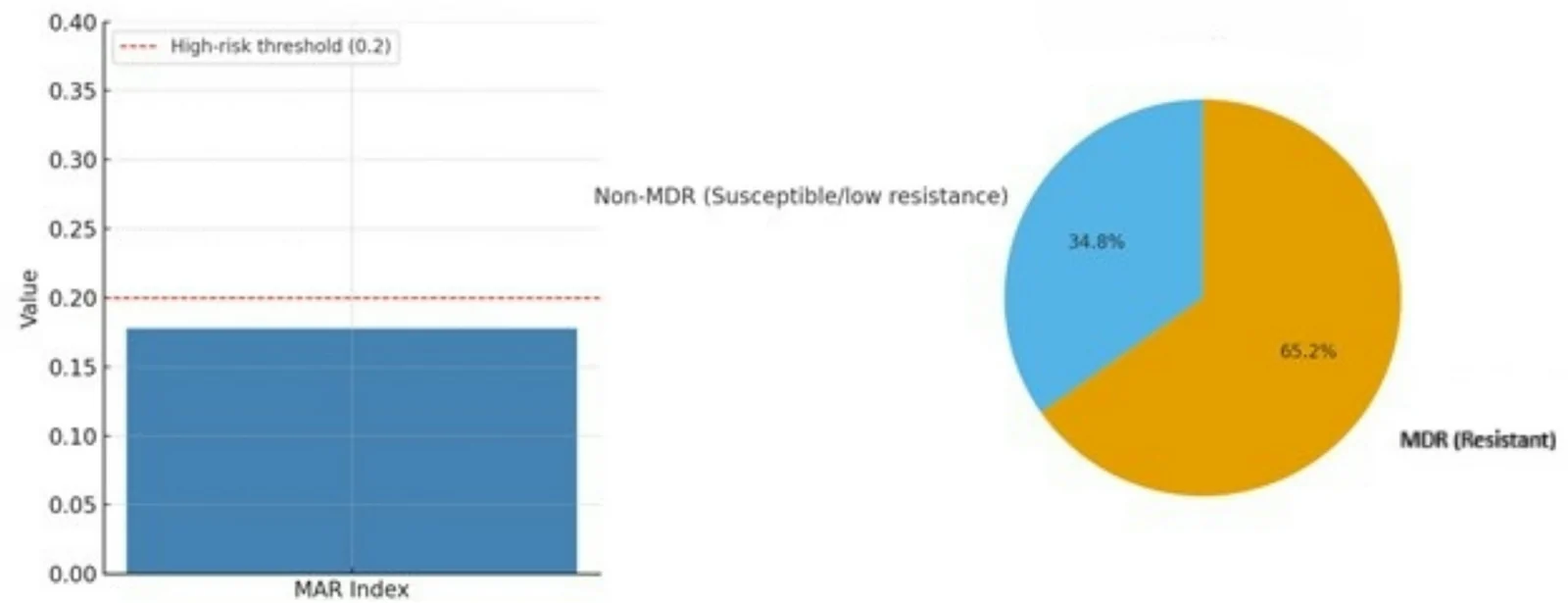
Graphical representation of MAR index and MDR status of the isolates

In the study, antibiotic susceptibility profiles of 115 *E. coli* isolates were also evaluated using descriptive statistics and ratio analysis (Fig. 3). The highest resistance rate was observed for the antibiotic cephalothin at 100%, followed by ampicillin at 25.21%. Resistance rates for other antibiotics were generally low and ranged from 0 to 9%. The combined report of both high resistance (25.21%) and high susceptibility (73.9%) for ampicillin may suggest heterogeneity among isolates. In particular, 100% resistance to cephalothin may indicate widespread and uncontrolled use of this antibiotic in veterinary field practice. The multiple antibiotic resistance (MAR) index was calculated for each isolate according to the method described by Krumperman (1983), using the formula a/b, where *a* represents the number of antibiotics to which the isolate was resistant and *b* represents the total number of antibiotics tested. Based on the commonly accepted threshold (MAR ≥ 0.2), isolates were categorized as multidrug-resistant (MDR) or non-MDR. As summarized in Fig. 4, a substantial proportion of the isolates (65.2%) were classified as MDR, indicating a considerable cumulative resistance burden at the population level.

**Fig. 5:**
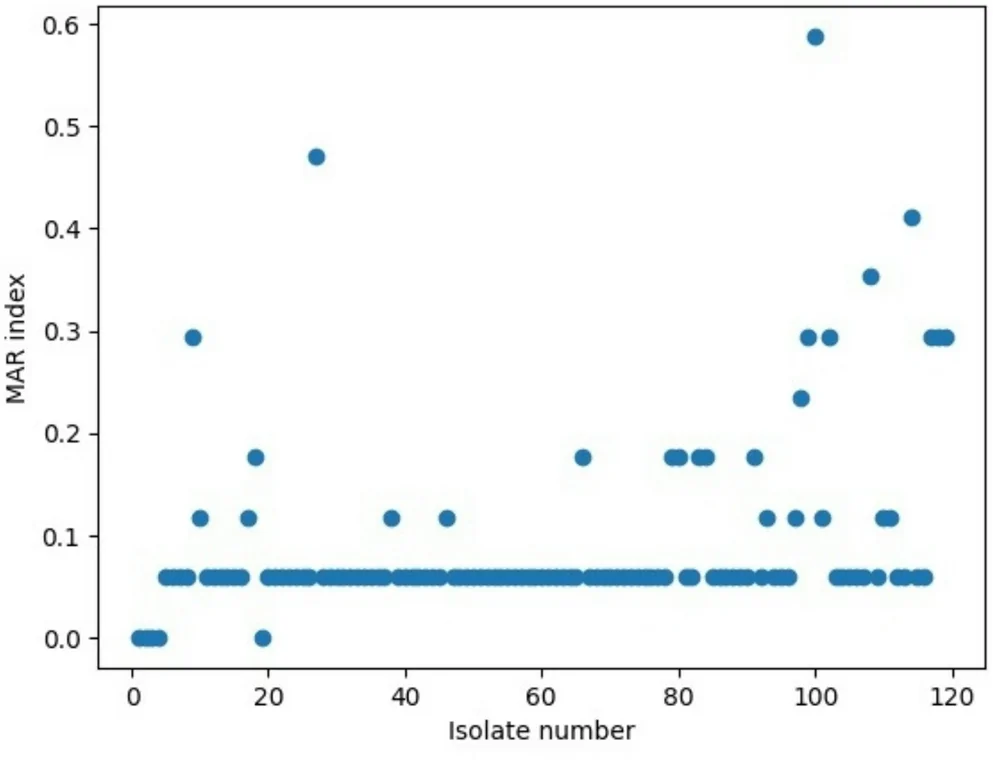
Distribution of multiple antibiotic resistance (MAR) indices among individual *E. coli* isolates (*n* = 115) recovered from raw milk samples

To further explore isolate-level variation underlying this MDR classification, the MAR index was additionally evaluated for each individual isolate. As shown in Fig. 5, MAR values varied widely across the 115 isolates, ranging from very low to relatively high levels. While many isolates exhibited low MAR indices, a distinct subset displayed elevated MAR values, reflecting a higher cumulative resistance burden. This distribution demonstrates pronounced heterogeneity among isolates and highlights that pooled MDR proportions may obscure isolate-specific resistance patterns. Overall, the antibiogram and MAR index analyses reveal that *E. coli* isolates obtained from raw milk samples exhibit heterogeneous but, in some cases, substantial resistance profiles, underscoring their potential relevance for public health and zoonotic risk.

### Detection and distribution of UPEC-associated virulence genes

**Fig. 6:**
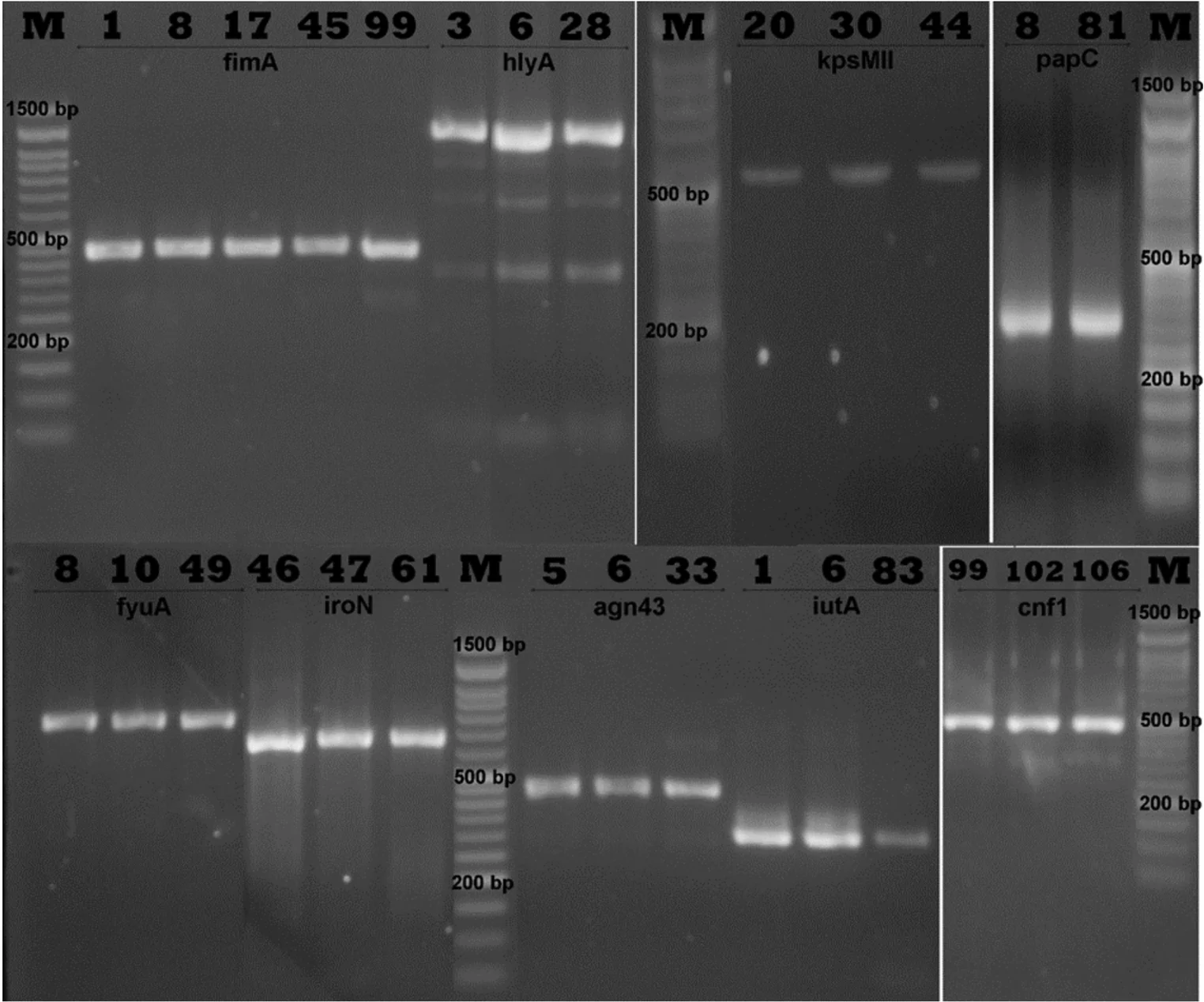
Gel electrophoresis images of UPEC-associated virulence genes. In the upper gel, strains positive for fimA (447 bp; lanes 1, 8, 17, 45, and 99), hlyA (1177 bp; lanes 3, 6, and 28), kpsMII (578 bp; lanes 20, 30, and 44), and papC (328 bp; lanes 8 and 81) are shown. In the lower gel, strains positive for fyuA (785 bp; lanes 8, 10, and 49), iroN (667 bp; lanes 46, 47, and 61), agn43 (433 bp; lanes 5, 6, and 33), iutA (302 bp; lanes 1, 6, and 83), and cnf1 (498 bp; lanes 99, 102, and 106) are shown. M indicates the molecular size marker (50 bp DNA Ladder RTU, GeneDireX, Taiwan)

In the study, UPEC virulence factors were amplified by the PCR method, and their presence in E. coli isolates was evaluated. A total of ten different virulence genes were analyzed, including hlyA, papC, cnf1, fimA, fyuA, vat, iroN, kpsMII, agn43, and iutA. The PCR amplification patterns of these genes are presented in Supplementary Figure S3, while representative amplification products from selected positive isolates are shown in Fig. 6. Distinct amplification profiles confirmed the presence of key virulence determinants among potential UPEC isolates; however, the vat gene was not detected in all isolates. The amplification pattern of the fimA virulence gene, a major adhesion factor contributing to bacterial attachment and biofilm formation, is shown in Supplementary Figure S4). The qualitative presence/absence profiles of individual virulence genes for each isolate are provided in Supplementary Table 2, while the overall detection frequencies are summarized in Table 4. As shown in Table 4, the most prevalent virulence genes were fimA (98.55%) and agn43 (95.65%). Iron acquisition–related genes were detected at moderate frequencies, including fyuA (31.88%), iutA (26.09%), hlyA (26.09%), kpsMII (11.59%), and iroN (8.70%). In contrast, papC (2.90%) and cnf1 (4.35%) were detected in a limited number of isolates, and the vat gene was not detected in any of the analyzed isolates. Evaluation of qualitative gene profiles based on Supplementary Table 2 demonstrated that the most frequent virulence gene combinations were dominated by adhesion-associated (fimA) and biofilm-related (agn43) determinants. These genes frequently co-occurred with one or more iron acquisition genes, including fyuA, iutA, or iroN. Several isolates carried up to six different virulence genes, whereas the majority of isolates harbored between two and four virulence-associated genes.

**Table 4:** Percentage of UPEC virulence genes in isolates

Virulence gene	Positive isolate (*n*)	Percentage (%)
*fimA*	68	98.55
*fyuA*	22	31.88
*hlyA*	18	26.09
*papC*	2	2.90
*cnf1*	3	4.35
*iroN*	6	8.70
*kpsMII*	8	11.59
*agn43*	66	95.65
*iutA*	18	26.09
*vat*	0	0.00

### Correlation analysis between antibiogram and UPEC-associated genes

**Fig. 7:**
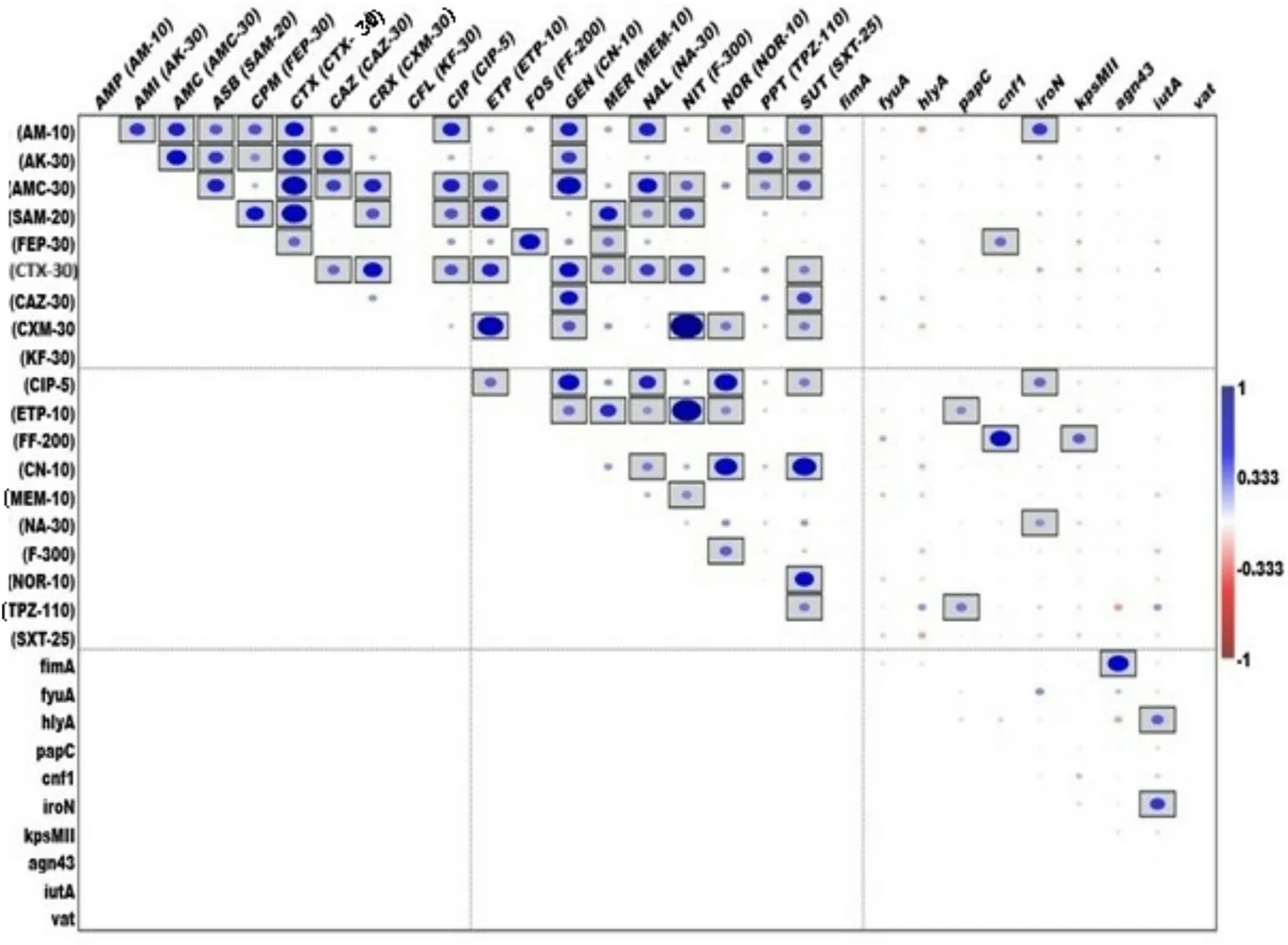
Pearson correlation analysis between antimicrobial resistance phenotypes (antibiogram profiles) and UPEC-associated virulence genes among *Escherichia coli* isolates. Correlations were calculated based on the presence or absence of resistance to individual antibiotics and virulence genes. Blue dots indicate positive correlations, whereas red dots indicate negative correlations, with dot size reflecting the strength of the correlation coefficient. Boxes denote statistically significant correlations (*p* < 0.05)

When the Pearson correlation relationship between the antibiogram of the 69 tested strains and the genes screened for UPEC is examined, it is seen that many antibiotics show a positive correlation with each other at a significance level of *p* < 0.05 (Fig. 7). When the correlation between antibiotics and UPEC-related genes was analyzed, no significant relationship was found between *fimA*, *fyuA*, *hlyA*, *agn43*, *iutA*, and *vat* genes and any antibiotics. In terms of correlation, very low negative or positive relationships were observed between these gene regions and antibiotics. A positive correlation was detected between *papC* and two antibiotics (ETP-10 and TPZ-110), *cnf1* and two antibiotics (FEB-30 and FF-200), *iroN* and three antibiotics (AM-10, CIP-5, and NA-30), and finally *kpsMII* and one antibiotic (FF-200) at the *p* < 0.05 level. In addition, a positive correlation was found between *fimA* and *agn43*, as well as *hlyA* and *iroN* and *iutA* at the *p* < 0.05 level.

### Correlation analysis of antibiotic resistance and virulence genes

In the study, we examined the relationships between antibiotic resistance profiles and some important virulence genes identified in UPEC strains, and very strong positive correlations were found (*r* > 0.8). These statistically significant correlations are summarized in Supplementary Table S3, which was derived from the Pearson correlation analysis presented in Fig. 7. The obtained correlation coefficients suggest that some virulence factors are co-carried on the same mobile genetic elements (e.g., plasmids, transposons) as resistance genes and spread together through co-selection. The correlation between amikacin (AK) and *hlyA* and *cnf1* genes was determined as *r* = 0.939 and *r* = 0.878, respectively. Correlations between cefepime (FEP) and cefuroxime (CXM) and *cnf1* were at *r* = 0.944 and *r* = 0.946, respectively, indicating that strains resistant to these two antibiotics carry the *cnf1* gene at a high rate. A high correlation (*r* = 0.872) was also detected between cephalexin (KF) and *iroN*, an important component of the siderophore system. The correlation coefficient between temocillin (TPZ) and the *kpsMII* gene involved in capsule biosynthesis was *r* = 0.975, which is the strongest relationship obtained in this study. A strong correlation (*r* = 0.805) was also observed between meropenem (MEM), a carbapenem group antibiotic, and *iroN*. The correlation between fluoroquinolones norfloxacin (NOR) and *iroN* (*r* = 0.901) indicates that genes related to iron metabolism may be associated with resistance acquisition in association with mutations in DNA gyrase/topoisomerase IV, which is an antibiotic target.

### Molecular identification of isolates by 16 S rRNA sequencing and BLAST analysis

16 S rRNA gene region was amplified from genomic DNA samples of potential *E. coli* isolates and the amplification products were analyzed by 1.5% agarose gel electrophoresis. Dense, single bands approximately 1.500 bp in size were observed in each well of each sample. These results demonstrate that the universal primer sets used specifically amplified the targeted 16 S rRNA gene region and that the PCR reactions were technically successful.

**Fig. 8:**
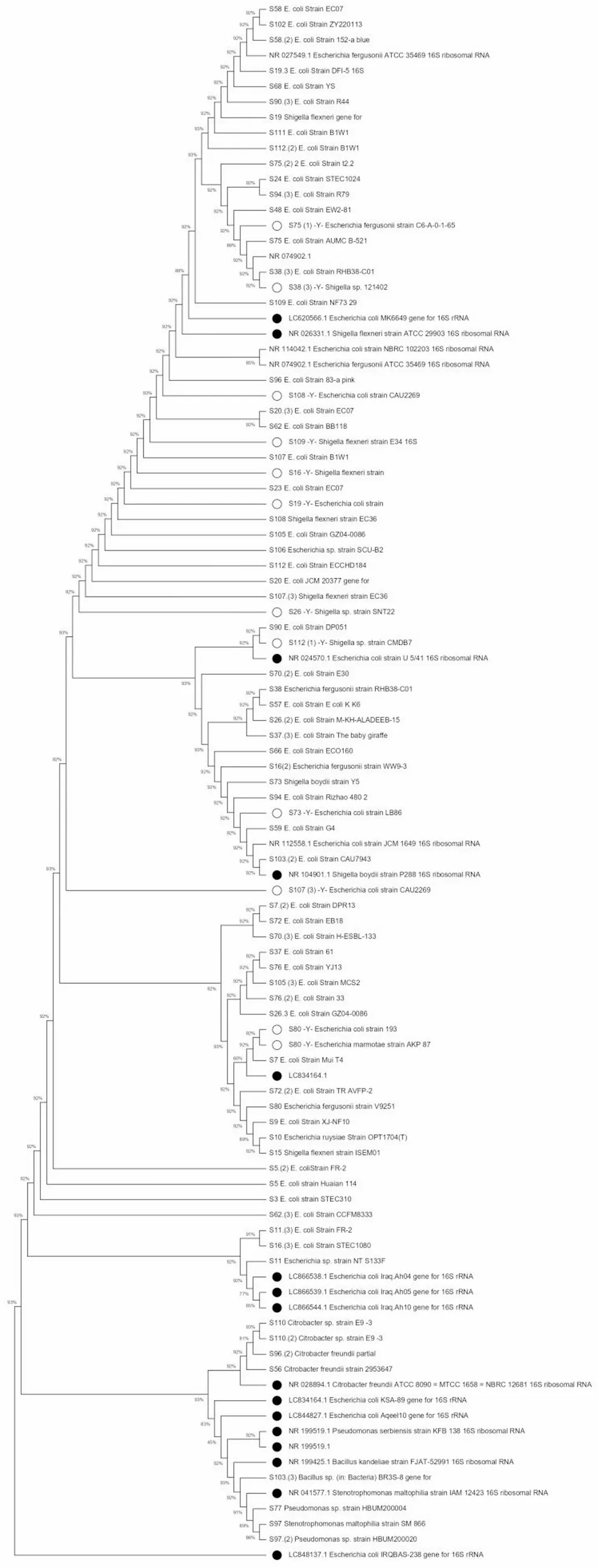
Phylogenetic tree of the isolates analyzed in the study* Evolutionary analysis with maximum likelihood method

**Fig. 9:**
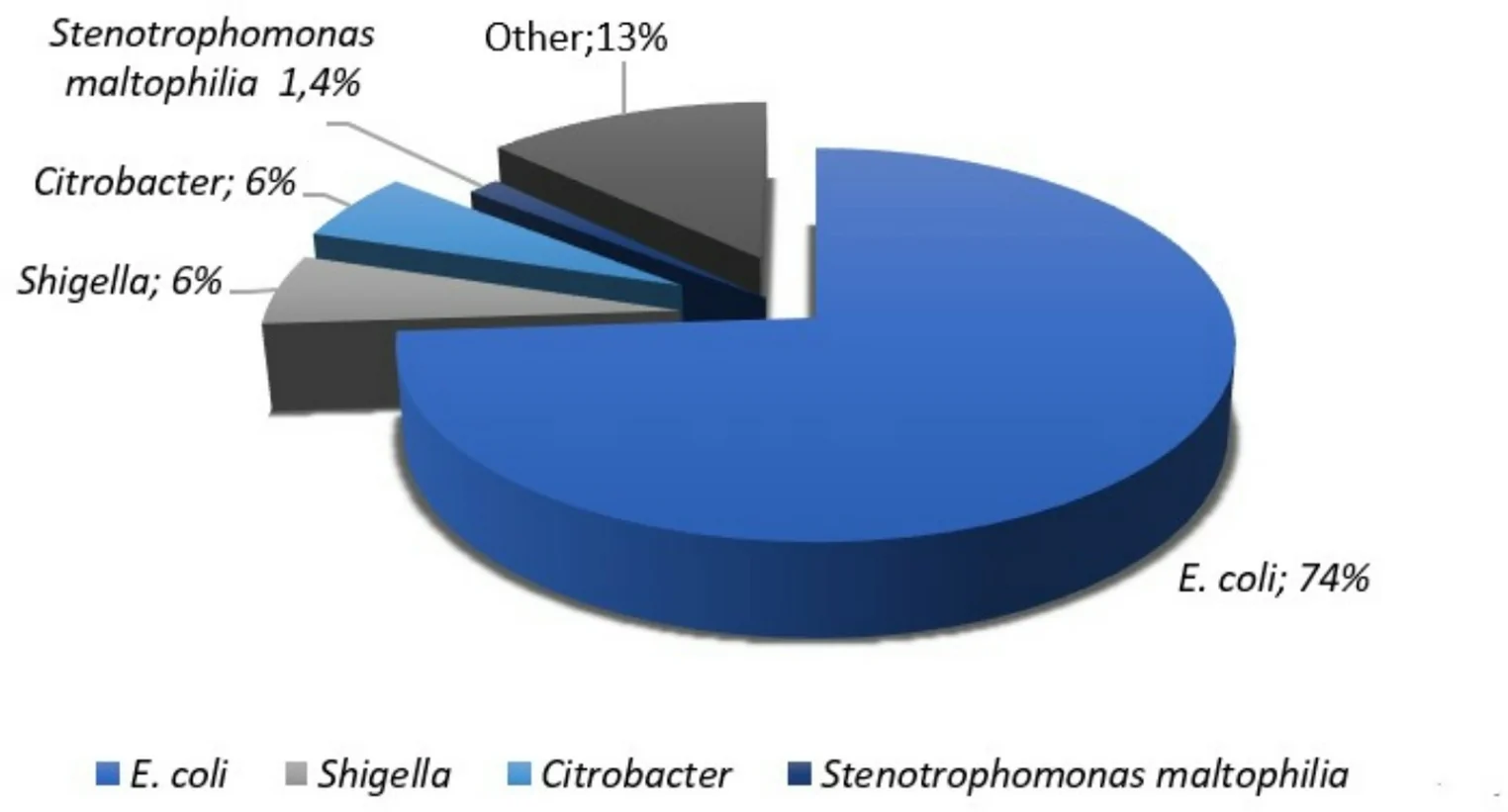
Species distribution of isolates based on BLAST analysis

16 S rRNA PCR products were sequenced using Sanger sequencing, and the resulting sequence data were compared with the NCBI database using Basic Local Alignment Search Tool (BLAST) analysis. The analyses revealed that the majority of the isolates matched reference strains of the *E. coli* species with high similarity rates (≥ 99%). In particular, ≥ 99% homology of the isolates with the 16 S rRNA gene regions of *E. coli* strains allowed these microorganisms to be identified as *E. coli* at the species level (Fig. 8). These results are consistent with the species identification threshold of 98% and above reported in the literature. Furthermore, the distribution of *E. coli*, *Shigella* sp., *Citrobacter freundii*, *Stenotrophomonas maltophilia*, and other species in the obtained matches was 74%, 6%, 6%, 1.4% and 13%, respectively (Fig. 9).

### Determination of UPEC potential by combined evaluation of sequencing and virulence gene analyses

Sequencing analyses of the 16 S rRNA gene revealed that the majority of the 69 isolates evaluated (*n* = 51) shared a high level of genetic similarity to *Escherichia coli*, ensuring species-level molecular identification accuracy. Simultaneous PCR analyses were conducted to investigate the presence of 10 UPEC-specific virulence genes, providing detailed molecular information regarding the potential pathogenicity of the isolates (Fig. 6).

This two-stage molecular analysis strategy applied to the isolates not only provided taxonomic confirmation but also enabled the assessment of each isolate’s uropathogenic potential. Based on the widely accepted criterion of carrying ≥ 3 virulence genes for UPEC diagnosis, 34 of the 51 *E. coli* isolates (66.60%) were classified as potential UPEC. The remaining 17 isolates were classified as non-UPEC due to inadequate virulence gene profiles. Potentially UPEC isolates were identified across all sampled regions, although the frequency of detection varied between locations.

## Discussion

IMVIC test data demonstrate that they are an effective first-line assay for the differential diagnosis of *E. coli*. This is consistent with previous studies using similar sample types. For example, the classical (++--, IMVIC) profile was detected in nearly all *E. coli* strains isolated from raw milk samples in China, and the diagnostic accuracy of biochemical tests was reported to be high (Liu et al. 2021). In another study conducted in Egypt, the majority of *E. coli* strains obtained from milk and dairy products were found to have the classical IMVIC profile (Ombarak et al. 2016). In studies conducted in South Africa, PCR confirmation was performed after the suspicious strains were eliminated with IMVIC tests, and it was emphasized that biochemical tests provide significant time and resource savings (Caine et al. 2014). In our study, IMVIC positivity rate covered approximately 64% (47/73) of milk samples and 55.8% (115/206) of the colonies. In the study, IMVIC tests were used as an efficient method in the initial screening and differentiation phases; the exclusion of isolates showing biochemical incompatibility in the diagnosis of *E. coli* contributed to a specific diagnosis. However, the data obtained also demonstrate the limitations of IMVIC tests, and it should be noted that molecular confirmation methods are complementary to this process.

The high overall antibiotic susceptibility rate (87–99%) found in this study is a significant finding demonstrating that *E. coli* strains originating from raw milk can still be effectively treated with many common antibiotics. However, comparison of resistance rates with the literature highlights regional differences. For example, another study reported higher resistance rates to ampicillin and sulfamethoxazole in broiler *E. coli* isolates (Jakobsen et al. 2010). This difference may be explained by variables such as geographic region, antibiotic use policies, and animal husbandry practices.

Ten UPEC virulence genes (*hlyA*, *papC*, *cnf1*, *fimA*, *fyuA*, *vat*, *iroN*, *kpsMII*, *agn43*, and *iutA)* were evaluated to determine potential UPEC isolates. *FimA* and *papC*, which are among the adhesin and colony-forming genes were the most frequent genes determined. *FimA* is one of the structural components of type 1 fimbriae and contributes to the bacterial infection capacity by enabling adhesion to bladder epithelial cells. This gene is commonly found in UPEC strains and was reported to play a significant role in lower urinary tract infections (Sarowska et al. 2019). *papC* is an adhesin gene involved in the formation of P fimbriae and has been associated with upper urinary tract infections, particularly pyelonephritis (Johnson 1991). *hlyA* gene, responsible for hemolysin production, was detected in some strains. This gene targets the cell membrane and induces the production of alpha-hemolysin, which causes lysis. *kpsMII* gene, associated with capsule synthesis, is involved in the formation of capsular structures, which increase the bacterial resistance to phagocytosis. The presence of this gene, in particular, suppresses the immune response, allowing the bacteria to evade host defense mechanisms. Previous studies have reported that this gene is more frequently found in *E. coli* strains associated with severe clinical conditions such as sepsis and pyelonephritis (Roberts 1996). Other important virulence factors identified in the study include the siderophore genes *fyuA*, *iroN*, and *iutA*, which mediate iron uptake. These genes enable the bacteria to efficiently utilize limited iron resources in host cells, providing an advantage during the infection process. It is reported in literature that these genes are highly prevalent in UPEC strains and their presence strongly correlate with virulence capacity (Johnson and Stell 2000). *Agn43* gene, responsible for autotransporter proteins, is associated with colony morphology, cell adhesion, and biofilm formation. The positivity of this gene in some strains supports the biofilm-forming potential of UPEC isolates (Zalewska-Pią Tek et al., 2015). The cytotoxic necrotizing factor 1 (*cnf1*) gene disrupts epithelial cell morphology by affecting intracellular actin structures, facilitating bacterial invasion. The presence of this gene is generally associated with more severe and invasive infections. Previous studies have reported *cnf1* positivity between 10% and 30% (Blanco et al. 2004) and our findings support this range.

The *vat* gene encodes a vacuolating autotransporter toxin that has been implicated in UPEC pathogenesis by inducing vacuole formation and epithelial cell damage, thereby contributing to host tissue injury and bacterial persistence. Nevertheless, UPEC pathogenesis is known to rely on a multifactorial and highly heterogeneous set of virulence determinants, rather than on a single conserved toxin (Subashchandrabose et al. 2013). Accordingly, the distribution of individual virulence genes, including *vat*, varies considerably among UPEC isolates from different sources. Positive PCR amplification of *vat* gene was not detected in any of the isolates evaluated in the study (0%). While some studies have reported *vat* positivity rates ranging from 10% to 30% among UPEC strains (Apostolou et al. 2001; Lucero et al. 2005), other investigations have described this gene as rare or completely absent in certain UPEC collections (Parham et al. 2005; Nichols et al. 2016). In this context, the absence of the *vat* gene in all isolates analyzed in the present study is not unexpected and is consistent with previous reports highlighting the heterogeneous nature of UPEC virulence repertoires. Our findings suggest that the raw milk–derived isolates examined here may belong to UPEC-related genotypes that do not rely on *vat*-mediated cytotoxic mechanisms. This observation further supports the concept that food-associated UPEC-like strains may harbor virulence profiles distinct from those of classical clinical UPEC isolates, as proposed within the framework of foodborne urinary tract infections (FUTIs) (Nordstrom et al. 2013; García et al. 2023).

The prevalence and antimicrobial resistance profiles observed in this study are broadly comparable to reports from other regions, while also reflecting clear geographical differences. Higher resistance rates to β-lactams and sulfonamides have been reported in *E. coli* isolates from dairy products in Europe and East Asia, largely attributed to intensive antimicrobial use in livestock production systems (Jakobsen et al. 2010; Liu et al. 2021). In contrast, the relatively high susceptibility rates observed here are consistent with findings from regions with more restricted antimicrobial use. Likewise, the distribution of UPEC-associated virulence genes in raw milk–derived *E. coli* varies across countries. Clinical isolates from North America and Europe typically harbor higher frequencies of invasion- and toxin-related genes, such as *papC*, *hlyA*, and *cnf1* (Spurbeck et al. 2012; Whelan et al. 2023), whereas food-associated isolates more frequently carry adhesion- and biofilm-related determinants, including *fimA* and *agn43*, similar to the patterns observed in this study (Ombarak et al. 2016). These differences highlight the influence of local agricultural practices and antimicrobial usage on both virulence and resistance profiles.

When all data are evaluated together, it is understood that more than one virulence gene can coexist in UPEC strains, increasing the pathogenic capacity of the bacteria. The most commonly detected gene in the virulence gene analysis was *fimA*. This gene was detected positive in all isolates (98.55%) except one. The second commonly detected gene was *agn43* (95.65%). These genes were followed by the less commonly detected genes such as *fyuA* (31.88%), *hlyA* (26.09%) and *iutA* (26.09%). The prevalence of *fimA* in UPEC from UTI patients changed between 100% and 76% (Abdul Raheem Hasan et al., 2021; Zamani and Salehzadeh 2018). The most frequent gene combination was reported as *fimA*-*agn43* in Mexican unpasteurised fresh cheeses similar to our results (Guzman-Hernandez et al. 2016). *fyuA* gene was present between 60.1% and 77% of the UPEC clinical isolates (Habibi et al. 2017; Rezatofighi et al. 2021). *hlyA* prevalence changed between 10% and 67.5% in UPEC from clinical samples (Valadbeigi et al. 2019; Helmy et al. 2023). *iutA* prevalence changed between 100% and 62.2% in UPEC isolated from clinical samples (Munkhdelger et al. 2017; Arafa et al. 2022). In general, the majority of isolates carried between two and four virulence genes. One of the commonly used criteria for UPEC identification is the presence of at least three different virulence genes (Brons et al. 2020; Derakhshan et al. 2022). UPEC highlight the distinctiveness of the coexistence of gene clusters corresponding to key pathogenicity functions such as adhesion, toxicity, and iron uptake (Spurbeck et al. 2012). In this study, 51 of the 69 isolates evaluated in terms of sequencing and virulence gene analysis were determined to belong to the *E. coli* species, and 34 of these isolates (66.60%) could be considered potential UPEC. The virulence gene distribution and co-occurrence patterns observed in this study differ markedly from those commonly reported for clinical UPEC isolates. Clinical strains, particularly those associated with upper urinary tract infections, frequently harbor combinations of adhesins and toxins such as *papC*, *hlyA*, and *cnf1*, reflecting an invasive pathogenic strategy (Stanley et al., 1998; Spurbeck et al. 2012; Whelan et al. 2023). In contrast, the raw milk–derived isolates analyzed here were dominated by adhesion- and biofilm-associated genes, most notably *fimA* and *agn43*, whereas invasion- and cytotoxicity-related determinants were detected at low frequencies.

The limited presence of *papC* and *cnf1* and the complete absence of *vat* further distinguish these isolates from classical clinical UPEC phenotypes. Conversely, the frequent co-occurrence of *fimA* and *agn43*, often accompanied by iron acquisition genes such as *fyuA*, *iutA*, and *iroN*, indicates a virulence profile oriented toward epithelial attachment, biofilm formation, and survival under iron-limited host conditions. Similar patterns have been reported for food-associated and zoonotic UPEC-like strains, which rely more on colonization and persistence than on acute invasive disease (Spurbeck et al. 2012; Flores-Mireles et al. 2015; Nordstrom et al. 2013). Beyond virulence gene composition, the clinical relevance of UPEC-like isolates is also influenced by their antimicrobial resistance profiles; therefore, correlations among antibiotic resistance patterns are evaluated.

The observation that some biochemically *Escherichia coli*–consistent isolates showed high 16 S rRNA sequence similarity to closely related taxa such as *Shigella* and *Citrobacter* reflects the well-recognized limitation of 16 S rRNA in resolving members of the *E. coli–Shigella* complex rather than true misidentification. Phylogenomic analyses have demonstrated that *Shigella* is genomically embedded within *E. coli* and is frequently regarded as an *E. coli* pathotype, explaining the limited species-level resolution provided by 16 S rRNA within this group (Yang et al. 2017). In this context, isolates exhibiting very high sequence similarity were interpreted at the *E. coli* species level for the purposes of this study.

When the correlation analysis between antibiotics considered, strong positive correlations were observed between AMP and antibiotics such as AMC and CTX (dark blue ellipses). Based on these data, it may be predicted that all three antibiotics generally undergo the same resistance mechanism (e.g., beta-lactamase production) and may thus induce cross-resistance. Significant positive correlations were found between CIP, NAL, and NOR, suggesting cross-resistance, which is common among quinolones, and the effectiveness of shared targets (e.g., DNA gyrase). Since DNA gyrase is an enzyme found only in prokaryotes and is vital for bacterial growth, it may be considered as an ideal target for quinolone antibiotics (Fàbrega et al. 2009). In *E. coli*, mutations occurring in the *gyrA* gene can significantly increase nalidixic acid resistance, while other mutations occurring in the *gyrA* or *topoisomerase IV* gene regions additionally lead to the development of high-level resistance to fluoroquinolones (Hopkins et al. 2005). Quinolone resistance can negatively affect the treatment process, and the spread of resistance genes through horizontal gene transfer can make the control of infectious diseases more complex. Positive correlations were also found between aminoglycosides such as GEN and KAN. These correlations are expected to be strong because of their common target, protein synthesis inhibition. Though antibiotic resistance can not be considered a direct virulence factor, it can have determinative effects on the development and course of infection under certain biological and clinical conditions. In particular, the ability of an antibiotic-resistant bacterial strain to effectively colonize specific anatomical sites within the host may lead to increased pathogenicity and treatment failure (Beceiro et al. 2013). In terms of infection dynamics, antibiotic resistance and virulence are complex interacting properties rather than two completely independent phenomena. Therefore, evaluation of both features together is of critical significance in understanding infections and developing targeted treatment strategies.

As detailed in the Results section, correlation analyses identified statistically significant and biologically interpretable associations between antimicrobial resistance and virulence gene profiles in UPEC strains, underscoring the interconnected nature of these traits. The predominance of genes such as *cnf1*, *hlyA*, *iroN*, and *kpsMII* in strains with high resistance profiles suggests that these pathogens exhibit both treatment resistance and invasiveness. This poses a serious threat in hospital-acquired infections and should be considered in determining antimicrobial treatment strategies.

Recent genomic and functional studies have provided robust evidence supporting the FUTI mechanism. Comparative phylogenomic analyses have demonstrated close genetic relatedness and shared virulence determinants between ExPEC isolates recovered from retail meat products—particularly poultry—and those causing human urinary tract infections, indicating common transmission lineages across food and clinical reservoirs (Johnson et al. 2005; Vincent et al. 2010; Liu et al. 2018; Manges et al. 2019; Guragain et al. 2025).

Importantly, poultry-derived ExPEC strains have been shown to adhere efficiently to bladder epithelial cells and to form strong biofilms, which are critical virulence traits facilitating urinary tract colonization and persistence, thereby confirming their functional uropathogenic potential (García et al. 2023). A schematic overview of the FUTI mechanism is provided in Supplementary Figure S1.

Together with molecular source-attribution studies demonstrating that a substantial proportion of community-acquired UTIs originate from food-animal reservoirs, these data firmly support the FUTI concept as a biologically plausible and epidemiologically relevant pathway of infection. Consequently, retail meat products and other animal-derived foods, including raw milk, should be considered potential contributors to intestinal colonization by uropathogenic *E. coli*, thereby increasing the risk of subsequent ascending urinary tract infections in humans (Aziz et al. 2024).

UPEC potential was determined by combined evaluation of sequencing and virulence gene analyses as detailed results are presented in the article. Based on the widely accepted criterion of carrying ≥ 3 virulence genes for UPEC diagnosis, 34 of the 51 *E. coli* isolates (66.60%) were classified as potential UPEC. In our study, the high prevalence of UPEC in *E. coli* isolates (66.60%) from raw milk indicates that dairy products can be a serious vector not only for microbial contamination but also for potentially virulent zoonotic strains. This finding highlights the potential for UPEC strains to be transmitted to humans through the food chain and poses a significant public health threat to food safety.

The findings of this study underscore the critical importance of hygiene practices throughout the raw milk production chain from a public health perspective. The detection of antimicrobial-resistant *Escherichia coli* and UPEC-associated virulence genes in raw milk isolates suggests that inadequate hygiene during milking, storage, and transportation may pose potential risks for contamination. In particular, the consumption of raw milk or insufficiently heat-treated dairy products may facilitate the transmission of resistant and potentially pathogenic bacteria to consumers via the food chain. These observations highlight the necessity of strict food safety measures as well as the rational and controlled use of antimicrobials in livestock production. Inappropriate or excessive antimicrobial use contributes to the selection of resistant bacteria, representing long-term risks to both animal and human health. In this context, improving hygiene standards in dairy production and strengthening antibiotic stewardship strategies are considered essential components for limiting the spread of foodborne antimicrobial resistance (WHO 2017; EFSA 2020).

## Conclusion

The comprehensive investigation of raw milk samples from rural Malatya revealed critical insights into the public health risks posed by *Escherichia coli* contamination, antibiotic resistance, and uropathogenic potential. Using an integrative workflow that combined phenotypic identification, molecular verification, antibiogram testing, and virulence gene profiling, 206 bacterial colonies were isolated, of which 115 were confirmed as *E. coli*. Antibiotic susceptibility testing demonstrated universal resistance to cephalothin, suggesting the presence of antimicrobial selection pressure that may be associated with antibiotic use in livestock production systems. The calculated Multiple Antibiotic Resistance (MAR) index of 0.178 reflected moderate antibiotic selection pressure among isolates.

Species-level confirmation via 16 S rRNA gene sequencing and BLAST analysis showed that 51 of 69 analyzed isolates (73.91%) shared ≥ 99% sequence homology with *E. coli* reference strains. Screening for 10 virulence genes revealed that 34 of the 51 confirmed isolates (66.60%) carried at least three virulence determinants, classifying them as potential uropathogenic *E. coli* (UPEC). These findings indicate that raw milk may act not only as a carrier of *E. coli* contamination but also as a potential reservoir for multidrug-resistant and uropathogenic strains.

From a public health perspective, raw milk may represent a potential exposure route when hygienic processing and handling conditions are insufficient. Strengthening microbiological surveillance, promoting rational antibiotic use, and implementing rigorous hygiene measures throughout the dairy production chain—from milking to retail—are essential to reduce foodborne transmission risks. Scientific data–driven training and monitoring programs will play a pivotal role in preventing infections, particularly urinary tract infections, and safeguarding public health.

Overall, these findings underscore the need for improved hygiene practices in dairy production and responsible antibiotic stewardship to reduce consumer exposure to resistant and potentially virulent *E. coli* strains.

Taken together, the results support the possibility that raw milk may serve as a potential reservoir of ExPEC strains carrying virulence traits associated with urinary tract infections; however, further studies are needed to better clarify this relationship. This study aligns with the United Nations Sustainable Development Goals (UN SDGs), particularly SDG 3 (Good Health and Well-Being) and SDG 12 (Responsible Consumption and Production). By addressing antimicrobial resistance and foodborne transmission pathways, the findings contribute to public health protection and support the development of safer and more sustainable food systems. Future studies incorporating whole-genome sequencing, functional virulence assays, particularly biofilm formation analyses, will be essential to further elucidate the pathogenic potential of raw milk–derived UPEC isolates.

## Supplementary Information

Below is the link to the electronic supplementary material.

Supplementary Material 1 (DOCX 34.8 KB)

Supplementary Material 2 (DOCX 691 KB)

## Data Availability

Data will be made available on request.
