# Supplementary material for: Prevalence, antibiotic resistance, and virulence gene profiles of uropathogenic Escherichia coli (UPEC) isolated from raw milk: implications for public health
Source: Folia Microbiol (Praha). 2026 Mar 26;71(5):1447–66. doi: 10.1007/s12223-026-01461-x (PMC13736335; doi:10.1007/s12223-026-01461-x)
Supplement: Supplementary file 1 [file 12223_2026_1461_MOESM1_ESM.docx]

**Prevalence, antibiotic resistance, and virulence gene profiles of uropathogenic *Escherichia coli* (UPEC) isolated from raw milk: implications for public health**

**Aynur Akan^1^, Seval Cing Yildirim^2*^ Cumhur Avsar^3^, Zeynep Yegin^4^**

^1^Department of Biology, Faculty of Arts & Sciences, Inonu University, Malatya, Turkey, <https://orcid.org/0009-0000-6138-9257>

^2^Department of Biology, Faculty of Arts & Sciences, Inonu University, Malatya, Turkey, <https://orcid.org/0000-0001-7163-9512>

^3^Department of Biology, Faculty of Arts & Sciences, Sinop University, Sinop, Turkey,

<https://orcid.org/0000-0002-4095-0022>

^4^Medical Laboratory Techniques Program, Vocational School of Health Services, Sinop University, Sinop, Turkey, <https://orcid.org/0000-0003-4637-0253>

*Corresponding author: Seval Cing Yildirim

Department of Biology, Faculty of Arts & Sciences, Inonu University, Malatya, Turkey

E-mail: [seval.cing@inonu.edu.tr;](mailto:seval.cing@inonu.edu.tr;) [scingy@gmail.com](mailto:scingy@gmail.com)

**Supplementary Table S1.** Functional classification and rationale for the selection of UPEC-associated virulence genes analyzed in this study*

| Gene | Virulence category | Gene product / Function | Role in UPEC pathogenesis | Rationale for selection |
| --- | --- | --- | --- | --- |
| fimA | Adhesion | Type 1 fimbrial major subunit | Mediates adhesion to uroepithelial cells and initiates colonization of the urinary tract | Essential for initial attachment and a hallmark virulence factor of UPEC |
| papC | Adhesion | P fimbrial assembly protein | Facilitates binding to kidney epithelial cells and is associated with pyelonephritis | Strongly linked to upper urinary tract infections and UPEC pathogenicity |
| agn43 | Biofilm formation | Antigen 43 (autoaggregation protein) | Promotes bacterial aggregation, biofilm formation, and persistence | Key determinant of biofilm-mediated survival and chronic infection |
| cnf1 | Toxin production | Cytotoxic necrotizing factor 1 | Induces host cell damage and modulates immune responses | Contributes to tissue injury and increased virulence in UPEC strains |
| hlyA | Toxin production | Alpha-hemolysin | Causes host cell lysis and inflammation | Major toxin associated with severe UPEC infections |
| fyuA | Iron acquisition | Yersiniabactin siderophore receptor | Mediates high-affinity iron uptake under iron-limited host conditions | Enhances extraintestinal survival and virulence during infection |
| iroN | Iron acquisition | Salmochelin siderophore receptor | Enables iron uptake while evading host immune sequestration | Frequently detected in extraintestinal pathogenic E. coli |
| iutA | Iron acquisition | Aerobactin siderophore receptor | Supports bacterial growth in iron-restricted environments | Associated with increased fitness and invasiveness of UPEC |
| kpsMII | Capsule formation | Group II capsular polysaccharide transport protein | Confers resistance to phagocytosis and serum killing | Capsule is a key factor in immune evasion and systemic spread |
| vat | Toxin / Autotransporter | Vacuolating autotransporter toxin | Induces cytotoxic effects and contributes to host tissue damage | Implicated in virulence of UPEC and other ExPEC strains |

* A synopsis of UPEC-associated virulence genes has been derived from Zhou et al., 2023.

**Supplementary Table 2**. Qualitative distribution of UPEC-associated virulence genes in raw milk–derived *E. coli* isolates

| **Isolates** | ***fimA*** | ***fyuA*** | ***hlyA*** | ***papC*** | ***cnf1*** | ***iroN*** | ***kpsMII*** | ***agn43*** | ***iutA*** | ***vat*** |
| --- | --- | --- | --- | --- | --- | --- | --- | --- | --- | --- |
| **1** | + | - | + | - | - | - | - | - | + | - |
| **2** | + | - | + | - | - | - | - | - | - | - |
| **3** | + | - | + | - | - | - | - | + | + | - |
| **4** | + | + | + | - | - | + | - | + | + | - |
| **5** | + | + | + | - | - | + | - | + | + | - |
| **6** | + | + | - | + | - | - | - | + | - | - |
| **7** | + | + | - | - | - | - | - | + | - | - |
| **8** | + | - | - | - | - | - | - | + | - | - |
| **9** | + | - | - | - | - | - | - | + | - | - |
| **10** | + | - | - | - | - | - | - | + | - | - |
| **11** | + | - | - | - | - | - | - | + | - | - |
| **12** | + | - | - | - | - | - | - | + | - | - |
| **13** | + | - | - | - | - | - | + | + | - | - |
| **14** | + | - | - | - | - | - | - | + | - | - |
| **15** | + | - | - | - | - | - | - | + | - | - |
| **16** | + | - | - | - | - | - | - | + | - | - |
| **17** | + | + | + | - | - | - | - | + | - | - |
| **18** | + | + | + | - | - | - | - | + | - | - |
| **19** | + | - | - | - | - | - | - | + | - | - |
| **20** | + | + | - | - | - | - | + | + | + | - |
| **21** | + | - | + | - | - | - | - | + | - | - |
| **22** | + | + | - | - | - | + | - | + | - | - |
| **23** | + | - | + | - | - | - | - | + | - | - |
| **24** | + | - | + | - | - | - | - | + | + | - |
| **25** | + | + | - | - | - | - | - | + | + | - |
| **26** | + | - | - | - | - | - | + | + | + | - |
| **27** | + | + | + | - | - | - | - | + | - | - |
| **28** | + | + | - | - | - | + | - | + | + | - |
| **29** | + | - | - | - | - | + | - | + | + | - |
| **30** | + | + | + | - | - | - | + | + | + | - |
| **31** | + | + | - | - | - | - | - | + | + | - |
| **32** | + | - | - | - | - | - | - | + | - | - |
| **33** | + | - | - | - | - | - | - | + | - | - |
| **34** | + | - | - | - | - | - | - | + | - | - |
| **35** | + | + | - | - | - | - | - | + | - | - |
| **36** | + | - | - | - | - | - | - | + | - | - |
| **37** | + | - | - | - | - | - | - | + | - | - |
| **38** | + | - | - | - | - | + | - | + | + | - |
| **39** | - | - | - | - | - | - | - | - | - | - |
| **40** | + | - | + | - | - | - | + | + | - | - |
| **41** | + | - | - | - | - | - | - | + | + | - |
| **42** | + | - | - | - | - | - | - | + | - | - |
| **43** | + | - | - | - | - | - | - | + | - | - |
| **44** | + | - | - | - | - | - | - | + | - | - |
| **45** | + | - | - | - | - | - | + | + | - | - |
| **46** | + | - | + | + | - | - | - | + | + | - |
| **47** | + | - | + | - | - | - | - | + | + | - |
| **48** | + | - | - | - | - | - | + | + | - | - |
| **49** | + | - | - | - | - | - | - | + | - | - |
| **50** | + | - | - | - | - | - | - | + | - | - |
| **51** | + | - | - | - | - | - | - | + | - | - |
| **52** | + | - | - | - | - | - | - | + | - | - |
| **53** | + | - | - | - | - | - | - | + | - | - |
| **54** | + | - | + | - | - | - | - | + | - | - |
| **55** | + | - | + | - | - | - | - | + | - | - |
| **56** | + | - | + | - | - | - | - | + | + | - |
| **57** | + | - | - | - | - | - | - | + | - | - |
| **58** | + | - | - | - | + | - | - | + | - | - |
| **59** | + | - | - | - | - | - | - | + | - | - |
| **60** | + | - | - | - | + | - | - | + | - | - |
| **61** | + | + | - | - | - | - | - | + | - | - |
| **62** | + | + | - | - | + | - | + | + | - | - |
| **63** | + | + | - | - | - | - | - | + | - | - |
| **64** | + | + | - | - | - | - | - | + | - | - |
| **65** | + | + | - | - | - | - | - | + | - | - |
| **66** | + | + | - | - | - | - | - | + | - | - |
| **67** | + | + | - | - | - | - | - | + | - | - |
| **68** | + | - | - | - | - | - | - | + | + | - |
| **69** | + | + | - | - | - | - | - | + | - | - |

(+) indicates the presence and (–) indicates the absence of the corresponding virulence gene.

**Supplementary Table S3**. Summary of statistically significant Pearson correlations between antibiotic resistance phenotypes and UPEC-associated virulence genes identified in this study.

| **Antibiotic** | **Virulence gene** | **r value** | **p value** | **Interpretation** |
| --- | --- | --- | --- | --- |
| Amikacin (AK) | hlyA | 0.939 | <0.05 | Strong positive correlation |
| Amikacin (AK) | cnf1 | 0.878 | <0.05 | Strong positive correlation |
| Cefepime (FEP) | cnf1 | 0.944 | <0.05 | Strong positive correlation |
| Cefuroxime (CXM) | cnf1 | 0.946 | <0.05 | Strong positive correlation |
| Cephalothin (KF) | iroN | 0.872 | <0.05 | Strong positive correlation |
| Piperacillin–tazobactam (TPZ) | kpsMII | 0.975 | <0.05 | Very strong positive correlation |
| Meropenem (MEM) | iroN | 0.805 | <0.05 | Strong positive correlation |
| Norfloxacin (NOR) | iroN | 0.901 | <0.05 | Strong positive correlation |

This table summarizes the statistically significant correlations derived from the Pearson correlation analysis presented in Figure 7. Only correlations with r > 0.8 and p < 0.05 are shown.
