# Supplementary material for: Prevalence, antibiotic resistance, and virulence gene profiles of uropathogenic Escherichia coli (UPEC) isolated from raw milk: implications for public health
Source: Folia Microbiol (Praha). 2026 Mar 26;71(5):1447–66. doi: 10.1007/s12223-026-01461-x (PMC13736335; doi:10.1007/s12223-026-01461-x)
Supplement: Supplementary file 2 [file 12223_2026_1461_MOESM2_ESM.docx]

**Prevalence, antibiotic resistance, and virulence gene profiles of uropathogenic *Escherichia coli* (UPEC) isolated from raw milk: implications for public health**

**Aynur Akan^1^, Seval Cing Yildirim^2*^ Cumhur Avsar^3^, Zeynep Yegin^4^**

^1^Department of Biology, Faculty of Arts & Sciences, Inonu University, Malatya, Turkey, <https://orcid.org/0009-0000-6138-9257>

^2^Department of Biology, Faculty of Arts & Sciences, Inonu University, Malatya, Turkey, <https://orcid.org/0000-0001-7163-9512>

^3^Department of Biology, Faculty of Arts & Sciences, Sinop University, Sinop, Turkey,

<https://orcid.org/0000-0002-4095-0022>

^4^Medical Laboratory Techniques Program, Vocational School of Health Services, Sinop University, Sinop, Turkey, <https://orcid.org/0000-0003-4637-0253>

*Corresponding author: Seval Cing Yildirim

Department of Biology, Faculty of Arts & Sciences, Inonu University, Malatya, Turkey

E-mail: [seval.cing@inonu.edu.tr;](mailto:seval.cing@inonu.edu.tr;) [scingy@gmail.com](mailto:scingy@gmail.com)

**Supplementary Figure S1.** Schematic representation of the proposed mechanism of foodborne urinary tract infections (FUTIs). Contaminated food sources, particularly poultry products and raw milk, may serve as reservoirs of extraintestinal pathogenic *Escherichia coli* (ExPEC). Following oral ingestion, ExPEC may establish intestinal colonization mediated by virulence factors such as *fimH* and *papC*, biofilm formation, and antimicrobial resistance determinants. Subsequent fecal–perineal transfer may facilitate ascending infection of the urinary tract, ultimately leading to community-acquired UTIs. Anatomical characteristics in females, including a shorter urethra and closer proximity of the urethral opening to the perineal region, may further increase susceptibility to infection. The schematic diagram was created using BioRender.com and further edited by the authors for scientific presentation.

**Supplementary Figure S2.** Uncropped gel image showing genomic DNA integrity analysis of *E. coli* isolates.

*(Gel image prepared by Cumhur Avşar, Zeynep Yegin, and Aynur Akan)*

**Supplementary Figure S3.** PCR amplification patterns of virulence genes detected in potential UPEC strains.

*(Gel image prepared by Cumhur Avşar, Zeynep Yegin, and Aynur Akan)*

**Supplementary Figure S4.** PCR amplification pattern of the *fimA* virulence gene detected in potential UPEC strains. *(Gel image prepared by Cumhur Avşar, Zeynep Yegin, and Aynur Akan)*
